# Evaluating the effects of inhibitory control training as a universal prevention strategy for internalizing complaints in middle childhood

**DOI:** 10.1371/journal.pone.0358051

**Published:** 2026-09-21

**Authors:** Elizabeth J. Edwards, Khanh Linh Chu, Annemaree Carroll, Emily Ross, Kristof Hoorelbeke, Annabel De Clercq

**Affiliations:** 1 School of Education, The University of Queensland, Brisbane, Queensland, Australia; 2 Department of Clinical Psychology, Ghent University, Ghent, Belgium; Qufu Normal University, CHINA

## Abstract

Internalizing complaints or inwardly directed psychological difficulties such as anxiety, worry and low mood can significantly affect children’s emotional well-being and daily functioning. If left untreated, these difficulties can persist into adulthood. Growing evidence has reported the critical role of cognitive control (ability to regulate attention and memory processes) in the development and maintenance of emotional problems such as anxiety. Cognitive control training has shown promise in reducing emotional vulnerability in adults and adolescents. However, studies with children are scant. The present study examined the potential of a gamified inhibitory control training as a universal prevention strategy aimed at reducing emotional vulnerability in children. Given the link between inhibitory control and math achievement (i.e., greater inhibitory control associated with higher math achievement), we also explored whether 15 sessions of daily training could improve math performance. Our randomized controlled trial, conducted in accordance with a pre-registered, published protocol, used a 2 (Group: Inhibitory control training group, *n* = 62; vs. Active control group, *n* = 61) × 4 (Time: pre- vs. post-training vs. 1-month vs. 3-month follow-up) design in a non-selected sample of Australian children aged 8–11 years (*N* = 123). Emotional, cognitive and academic measures were collected at each timepoint. Intention-to-treat analyses using linear mixed models showed no evidence of proximal transfer to untrained inhibitory control measures and limited evidence of beneficial distal transfer to emotional or academic outcomes, with some outcomes showing trends favouring the active control condition. The absence of broader emotional and academic benefits may therefore reflect the lack of improvement in the targeted cognitive process itself. These findings highlight important considerations regarding the design and delivery of cognitive training interventions to universal samples, including the calibration of task demands and the conditions required to support cognitive transfer. Future research should investigate whether stronger transfer effects can be achieved through adaptive training tasks that maintain optimal cognitive challenge, extended or higher-dose training protocols, and interventions targeting children with elevated emotional vulnerability or inhibitory control difficulties.

## Introduction

Anxiety disorders rank among the most prevalent mental health problems during middle childhood, affecting 6–10% of children globally and substantially impacting daily functioning [1]. Without timely intervention, anxiety symptoms in children can persist into adolescence and adulthood, and are associated with poor academic outcomes, social impairments, and elevated risk for comorbid mood disorders [2,3]. Psychological interventions for childhood anxiety are typically costly and resource-intensive (e.g., one-on-one therapy), and access is hindered by long waitlists in healthcare systems [4]. These barriers highlight the urgent need for scalable and accessible approaches to reduce the treatment gap (i.e., the disparity between the number of children experiencing anxiety and those receiving support) [5]. Schools represent a unique context for delivering universal anxiety interventions which can be embedded within daily routines, minimise stigma, and improve access [6,7]. Universal prevention approaches may be particularly valuable in school settings because they can strengthen protective cognitive and emotional processes prior to the onset of clinically significant symptoms, while avoiding some of the stigma and access barriers often associated with selective interventions [5]. One promising approach is cognitive control training, which aims to strengthen executive processes involved in the regulation of attention and emotion. Because anxiety has consistently been linked to disruptions in cognitive control, enhancing these processes may reduce emotional vulnerability before clinically significant symptoms emerge. Accordingly, the present study examined whether gamified inhibitory control training, delivered to a universal sample of children in school settings, could improve cognitive control, reduce emotional vulnerability, and enhance mathematics achievement.

The rationale for cognitive control training as a universal anxiety intervention is based on evidence that deficits in cognitive control may increase vulnerability to the development and maintenance of anxiety [8,9]. Individuals with poorer cognitive control may be less able to inhibit threat-related thoughts, disengage from negative information, or regulate emotional responses, thereby increasing vulnerability to anxiety [8]. Strengthening cognitive control has the potential to enhance top-down regulation of attention and emotion and reduce susceptibility to anxiety before clinically significant symptoms emerge. Attentional control theory provides a theoretical explanation for this relationship by proposing that individuals with poorer attentional control are less able to regulate attention in the presence of threat, increasing reliance on stimulus-driven processing and reducing goal-directed control. These processes contribute to the persistence of anxiety while also explaining why anxiety further disrupts cognitive control [10]. According to the unity and diversity model of executive function, anxiety-related interference can compromise attentional and broader cognitive control, manifesting as impairments in inhibitory control (suppressing automatic or dominant responses), shifting (switching flexibly between tasks or mental sets), and updating (monitoring and revising working memory contents) [11]. From here, we use the term *cognitive control* to refer to executive processes involved in goal-directed behaviour, including inhibitory control, shifting, and updating of working memory.

The last decade has seen an increase in research using cognitive control training as an intervention for reducing anxiety and emotional correlates such as worry, depression and rumination [12,13]. Cognitive control training refers to systematic, repeated practice on tasks designed to strengthen the executive processes that regulate goal-directed thought and behaviour, such as inhibitory control, shifting, and updating in working memory [14]. Grounded in theories of executive function [11] and cognitive control [10], cognitive control training interventions aim to enhance the efficiency and coordination of top–down regulatory mechanisms by targeting specific component processes [15]. Strengthening these executive processes is hypothesized to improve the regulation of attention and emotion regulation by increasing individuals’ capacity to inhibit threat-related thoughts, disengage from emotionally salient information, and maintain attention on goal-directed tasks. In turn, enhanced cognitive control may reduce cognitive vulnerabilities associated with anxiety, including excessive worry, rumination, and attentional biases towards threat, thereby providing a plausible pathway through which improvements in cognitive functioning transfer to emotional outcomes [8,10]. Training paradigms often employ adaptive difficulty, immediate feedback, and high trial repetition to promote neurocognitive plasticity, with the expectation that improvements in cognitive control will generalise beyond the trained tasks to untrained cognitive functioning and through enhanced attentional and emotional regulation, contribute to reduction in emotional vulnerability and improvements in everyday functioning [16,17].

There is growing evidence that daily cognitive control training can improve mood and reduce emotional vulnerability in adults [18–22] and adolescents [23,24]. However, there is limited research testing the efficacy of cognitive control training for the relief of anxiety and related internalizing symptoms in younger children. We conducted a systematic review of cognitive control training studies for reduction of anxiety and depression in children and adolescents [25]. Five studies used children in middle childhood (aged under 12 years). The review revealed that training targeting executive functions (inhibitory control, shifting, updating) demonstrated efficacy in improving cognitive control and reducing emotional symptoms and that 11–15 sessions of cognitive training delivered in schools was required to detect changes.

In the current study, we focused on training inhibitory control, a cognitive process which numerous behavioural and neurocognitive studies have linked to elevated anxiety, worry [26] and depression [27] in adults. However, the evidence for the link between anxiety and inhibitory control in children is inconclusive. For example, one study found no differences in inhibitory control as a function of anxiety among children aged 10–12-years [28] when anxiety was measured with the internalising subscale of the Child Behaviour Checklist [29]. Another study reported similar null findings in children aged 8–17 years [30] when anxiety was measured using the State-Trait Anxiety Inventory for Children [31]. Yet, it is possible that broad screening instruments, which capture general internalising or trait/state anxiety symptoms, lack the sensitivity to detect subtle variations in anxiety presentation in children [32]. Notwithstanding known age-related developmental improvements in inhibitory control across middle childhood [33], one 7-year longitudinal study found that inhibitory control deficits in children were an early vulnerability factor for the development of anxiety symptoms when assessed using semi-structured diagnostic interviews aligned with DSM criteria [34]. In addition to its empirical and theoretical relevance, inhibitory control was selected as the primary training target for three reasons. First, inhibitory control is considered a core component of cognitive control that underpins the regulation of attention and emotion, making it a plausible mechanism for reducing internalizing symptoms. Second, inhibitory control shows rapid developmental change during middle childhood, suggesting it may be particularly amenable to intervention during this period. Third, compared to working memory or multi-component executive function training, inhibitory control can be more precisely targeted using well-validated paradigms (e.g., Go/No-Go), allowing clearer interpretation of training and transfer effects.

Studies investigating the efficacy of cognitive control training interventions for reducing or preventing emotional symptoms in adolescents have usually targeted working memory updating [23,35,36]. Most studies in middle childhood have used commercial working memory training packages which train a mixture of cognitive processes concurrently [37,38]. To the best of our knowledge, only two studies have investigated the efficacy of inhibitory control training for reduction in emotional symptoms in children, with conflicting results. Shanok et al. [39] deployed an adaptive Go/No-Go task with a sample of children aged 8–12 years. Children were asked to undertake 16 training sessions (12–15 min. each) over 4 weeks. Compared to waitlist controls, children in the inhibitory training group reported reductions in anxiety (lower sores on Screen for Child Anxiety Related Disorders [40]) and depression (lower scores on Children’s Depression Inventory [41]) symptoms and mixed improvements in inhibitory control (i.e., near transfer; better effectiveness on Go/No-Go and Flanker, but not Stroop, and better efficiency for Flanker but not Go/No-Go or Stroop), from pre- to post-training [39]. Although these results are promising, the study was limited by a small pre-test sample size (*n* = 42) and a 25% attrition from pre- to post-training, suggesting some children may have disengaged from the training. Moreover, a key limitation of this study was the use of a waitlist control condition, which restricts the ability to attribute observed benefits to the specific mechanisms of the intervention, as non-specific factors such as expectancy, attention, and engagement were not controlled. Furthermore, the absence of follow-up tests prevents conclusions about the durability of effects beyond the training period. Ganesan et al.[42], investigated changes in internalizing symptoms, captured using the Strengths and Difficulties Questionnaire [43], following gamified inhibitory control training in a sample of 6–13-year-old children who were asked to train daily for 8 weeks (15 min, each). Contrary to their hypothesis, children in the experimental group who trained using the gamified Stop Signal task showed no difference in emotional symptoms immediately post-training or at 1-year follow-up, compared to those in a control group who trained using a gamified response speed task. They noted children in the experimental group showed greater inhibitory control (near transfer) pre-post, but not shifting or working memory (far transfer), compared to controls who showed no change. Ganesan et al. improved on earlier work with a larger sample (*n* = 235), included an active control condition, and follow-up testing. However, it is possible that the use of broad screening measures like the Strengths and Difficulties Questionnaire may have limited sensitivity to detect small or domain-specific changes in internalizing symptoms (e.g., anxiety, depression), particularly in non-clinical samples. Taken together, the inconsistent findings across studies [39; 42] warranted further research to examine the mechanisms and boundary conditions under which inhibitory control training may influence emotional outcomes in children, including factors such as task design, training dose, adherence, and outcome measurement.

In addition to studies investigating effects of cognitive control training on emotional symptoms, several studies have found improvements in broader indicators of functioning. In a study with adolescents, Beloe and Derakshan [23] reported reduced anxiety and depression, and gains in reading performance following working memory training. Studies in middle childhood have reported benefits for mathematics achievement following working memory training [44; 45], and commercial executive function training targeting inhibitory control, shifting, and working memory [17], although another study found no improvements in math achievement following inhibitory control training [46]. These studies, nonetheless, did not assess emotional outcomes. Given that inhibitory control is a key predictor of mathematics success in middle childhood [47], it is plausible that training inhibitory processes during this developmental period may also enhance mathematics performance. The relationship between inhibitory control and mathematics achievement is theorised to operate through several cognitive mechanisms, including the suppression of irrelevant information, maintenance of task goals, and regulation of impulsive responding during mathematical problem solving [46,47]. Recent meta-analytic evidence indicates that inhibitory control is positively associated with mathematics performance in elementary school children [47], while intervention studies suggest that executive function and inhibitory control training may support aspects of mathematical learning and reasoning [44–46]. At the same time, inhibitory control may also influence mathematics achievement indirectly through emotional pathways, for example by reducing anxiety or math anxiety that can interfere with cognitive performance. Accordingly, the present study explored mathematics achievement as a broader indicator of functional transfer, while recognising that direct cognitive and indirect emotional pathways may both contribute to any observed effects. To our knowledge, no study has simultaneously examined the effects of targeted inhibitory control training on anxiety and related correlates and mathematics achievement in middle childhood.

## The current study

The primary aim of the current study was to examine the efficacy of gamified inhibitory control training, delivered to a universal sample of children in school settings, to improve cognitive processes and reduce emotional vulnerability (i.e., anxiety, depressive symptoms and emotional correlates) in middle childhood. To control for limitations noted in the current literature we employed an active control training condition, gamified training tasks and monitoring strategies to enhance engagement and adherence and included follow up assessment of outcomes. We used a 2 (Group: Inhibitory control training vs Active control) × 4 (Time: pre- vs post-training vs 1-month vs 3-month follow-up) randomized design. Children in both groups trained for 10 minutes each day for 3 weeks (15 sessions) as part of their daily classroom routine. Changes in the cognitive (inhibitory control, shifting, updating), emotional (anxiety, worry, depression, rumination, math anxiety) and academic outcome measures (math achievement) were captured using valid, reliable, psychometric scales and tasks and compared at pre-, post-training, 1- and 3-month follow-up. Follow-up intervals were consistent with those used to assess maintenance and early decay in other cognitive training studies [23,24]. To improve engagement with training and minimise attrition, we embedded game elements in a Go/No-Go task (inhibitory control training task) and a visual identification task (active control training task). Both tasks were adaptive (i.e., the difficulty level was adjusted based on individual task performance) to account for differences in performance and provided immediate feedback for motivation.

We hypothesized that inhibitory control training would lead to near (i.e., improvement in untrained inhibitory control) and far cognitive transfer (i.e., improvements in shifting and updating) compared to active controls across the four timepoints (H1). Next, we predicted that children in the inhibitory control training group would report fewer internalizing complaints, namely anxiety, worry, depression, rumination, and maths anxiety, compared to those in the active control group, from pre- to post-training, with sustained effects at 1-month and 3-month follow up (H2). Finally, we hypothesized that children in the inhibitory control training group would demonstrate greater improvements in math achievement compared to active controls across the four timepoints (H3). We first evaluated effects of inhibitory control training on the outcome measures using intention-to-treat (ITT) analyses. Next, we conducted exploratory follow-up analyses on participants completing at least 11 training sessions (i.e., per-protocol analyses) [48].

## Method

### Design and ethics approval

We registered our 2 (Group: Inhibitory control training vs Active control) × 4 (Time: pre-training vs post-training vs 1-month follow-up vs 3-month follow-up) single-blind randomized study on the Open Science Framework (https://osf.io/de2qa). The complete protocol was submitted for peer-reviewed publication prior to data collection [46]. The study was approved by The University of Queensland Human Research Ethics Committee (2023/HE000462). Gatekeeper approval was received from participating Education departments and schools. Written informed consent was obtained from all parents and participating children. The study was conducted in accordance with the ethical principles of the Declaration of Helsinki (2013) and complies with the standards for medical research involving human subjects. Recruitment commenced on 1 September 2023, and data was collected between 10 February and 26 November 2024.

### Participants

One hundred and twenty-three typically developing children were recruited from Year 4 and Year 5 in four primary schools in Southeast Queensland, Australia. In line with the study protocol [48], all 123 randomized participants were included in ITT analyses. Both the inhibitory control training and active control groups did not significantly differ in terms of baseline demographics, emotional, cognitive, or academic measures (see Table 1). As can be seen, consistent with the universal prevention design, the sample was predominantly non-clinical. At baseline, 13.01% (*n* = 16) of the 123 participants exceeded the clinical cut-off for elevated anxiety symptoms, and 19.51% (*n* = 24) exceeded the cut-off for elevated depression, on their respective scales (see Emotional Measures). A descriptive table with the mean outcome per group per timepoint for the ITT population can be found in the supplementary materials (Supplemental Table 1 in S1 File).

**Table 1:** Participant Characteristics Across Training Groups at Baseline (Pre-Training).

	Inhibitory Control Training*n* = 62	Active Control*n* = 61		
	*Mean (SD) / n (%)*	*Mean (SD) / n (%)*	*t/χ2*	*p*
**Demographic**				
Age in years	9.77 (0.78)	9.79 (0.82)	0.09	.930
Sex			0.08	.784
Male	31 (50.00%)	33 (54.10%)		
Female	31 (50.00%)	28 (45.90%)		
**Emotional measures**				
Anxiety	25.90 (18.28)	33.47 (23.50)	1.97	.051
Worry	16.30 (8.92)	17.47 (9.16)	0.71	.477
Depression	8.53 (6.33)	10.84 (7.47)	1.84	.068
Rumination	13.34 (10.00)	15.33 (10.23)	1.09	.278
Math Anxiety	9.26 (4.15)	9.61 (4.41)	0.44	.658
**Cognitive measures**				
Inhibitory Effectiveness	2.59 (0.71)	2.59 (0.88)	0.01	.988
Inhibitory Efficiency	4.37 (1.28)	4.45 (1.76)	0.27	.790
Shifting Effectiveness	63.26 (18.70)	64.49 (21.06)	0.34	.736
Shifting Efficiency	52.29 (23.20)	48.17 (26.38)	−0.91	.367
Updating Effectiveness	1.15 (0.71)	1.06 (0.72)	−0.71	.479
Updating Efficiency	1.28 (0.91))	1.42 (0.96)	−0.84	.403
**Math achievement measure**				
Math Achievement	490.55 (13.24)	488.87 (16.76)	−0.61	.541

*Note.* Mean (SD) / n (*%*). Efficiency measures are multiplied by 1,000 to improve interpretability.

We used individual-level randomization stratified by class, such that, within each class, students were randomized to one of two training groups. Randomization was conducted separately for each class across the four schools, using a computer-generated random number sequence (https://www.randomizer.org/).

### Apparatus and material

Data collection and cognitive training were conducted on project-dedicated iPads: 9th generation, iOS 16, 64GB; Apple Inc. Emotional measures were collected using online questionnaires administered on Qualtrics (https://www.qualtrics.com). Cognitive measures were collected using computerized tasks administered using Inquisit software (https://www.millisecond.com/). Mathematics assessments were conducted using pencil and paper tests. Training was delivered using an adaptive Go/No-Go (Inhibitory control training group) or a visual identification task (Active control group), built for the purpose of this study using Gorilla Experiment Builder (https://gorilla.sc/). Both training tasks incorporated game elements (i.e., narrative, characters, token-based rewards) to enhance engagement and motivation.

### Emotional measures

**Anxiety.** Anxiety was measured using the Spence Children’s Anxiety Scale [49]; a 38-item self-report assessment of anxiety in children aged 8−15 years, including items to capture symptoms of social phobia, panic or agoraphobia, generalized anxiety, obsessive-compulsive, separation anxiety, and physical injury fears. Children responded using a 4-point Likert scale from 0 = *never* to 3 = *always.* Total anxiety was calculated by summing responses to all 38 items, with possible scores ranging from 0 to 114. Higher scores represent higher symptoms of anxiety. Other studies have reported very high internal consistency (α = .87−.94; [50,51]). In the present sample, internal consistency was excellent at four timepoints: pre: α = .92; post: α = .91; 1-month: α = .92, 3-month follow-up: α = .92.

**Worry.** Worry was assessed using the Penn State Worry Questionnaire for Children [52]; a 14-item questionnaire that measures the tendency to engage in excessive, generalised, and uncontrollable worry in children aged 7−17 years. Items were rated on a 4-point Likert scale from 0 = *never* to 3 = *always*. Total worry was calculated (after reversing 3 items) by summing the scores on all items with possible total worry scores ranging from 0 to 42. Higher scores indicate greater tendency to worry. Previous work has reported acceptable to excellent internal consistency (α = .70−.95; e.g.[52; 53]). In the present sample, internal consistency was excellent at four timepoints: pre: α = .91; post: α = .89; 1-month: α = .92, 3-month: α = .91.

**Depression.** Depression was indexed by the low mood subscale from the Revised Children’s Anxiety and Depression Scale [54]; a self-report assessment of symptoms of major depressive disorder in children aged 8−18 years. The low mood subscale included 10 items and children responded using a 4-point Likert scale from 0 = *never* to 3 = *always*. Possible total depression scores range from 0 to 30 with higher scores indicative of greater depressive symptoms (lower mood). Previous studies have reported acceptable to good internal consistency for the low mood subscale (α = .72−.82; e.g.[55],). In the present sample, internal consistency was good at four timepoints: pre: α = .86; post: α = .89; 1-month: α = .90, 3-month: α = .89.

**Rumination.** The rumination subscale from the Child Response Style Questionnaire [56] was used to capture repetitive negative thinking or rumination. Children responded to 13 items on a 4-point Likert scale from 0 = *almost never* to 3 = *almost always*. Possible total rumination scores range from 0 to 39 with higher scores representing greater rumination. Acceptable internal consistency has been reported (α = .78; [56]) and in the present sample, internal consistency was excellent at four timepoints: pre: α = .92; post: α = .91; 1-month: α = .94, 3-month: α = .94.

**Math Anxiety.** Math anxiety was indexed using the Math Learning Anxiety subscale from the Modified Abbreviated Math Anxiety Scale [57]. Children responded to five statements asking how anxious they would feel during certain situations involving math using a 5-point Likert scale from 1 = *low anxiety* to 5 = *high anxiety*. Possible Math Learning Anxiety scores range from 5 to 25 with higher scores showing greater anxiety about math. Previous work has reported acceptable to good internal consistency (α = .77−.80) (e.g.[57,58],). In the present sample, internal consistency was good at four timepoints pre: α = .81; post: α = .85; 1-month: α = .90, 3-month: α = .89.

### Cognitive measures

**Inhibitory Control.** A computerized child adaption of the standard (i.e., nonadaptive) Go/No Go task [59] was used to measure inhibitory control, namely the Whack a Mole task. Children were required to view visually presented stimuli (i.e., cartoon mole and cartoon eggplant) and respond, as quickly and accurately as possible, based on instructions, e.g., *Press the Spacebar when the mole appears on the screen* (Go) *but don’t press the Spacebar when you see an eggplant* (No-Go). The mole appeared with varied disguises (e.g., with hair, glasses, hat), but the eggplant was constant throughout the task. The task commenced with a practice block of 10 trials, followed by four test blocks ranging from 54 to 56 trials each (total of 220 test trials). There was a ratio of 75% Go to 25% No-Go trials across the test blocks. Trials started with the image of an empty mole hole appearing for 500 ms in the centre of the black screen. Go and No-Go stimuli were presented for a maximum of 1800 ms and 1300 ms, respectively. Visual feedback was given for 300 ms after each correct (i.e., *WACK!* for hitting the mole; *AWESOME* with a hand-clapping image for avoiding the eggplant) and incorrect response (i.e., *OOPS!* for missing the mole or whacking the eggplant). The total trial duration including feedback was 2300 ms. Following Edwards et al. [60], inhibitory effectiveness was operationalized using the discrimination sensitivity (*d*’): *z-*score of correct Go trials minus *z-*score of No-Go errors [61]. Inhibitory efficiency was operationalized as *d’* divided by mean RT on correct Go trials. Higher inhibitory effectiveness reflects better discrimination between Go and No-Go stimuli (fewer No-Go errors relative to correct Go responses). Inhibitory efficiency incorporates both accuracy and speed, with higher values indicating faster, more accurate responses. To avoid small numbers and aid interpretability, inhibitory efficiency was multiplied by 1,000 prior to the analysis.

**Shifting.** A Colour Shape-Shifting task [62] captured the ability to mentally shift between the demands of a task. Children were presented with two shapes (triangle, circle) coloured either red or green. Children were instructed to sort the stimuli by shape or by colour, as quickly and accurately as they could. They responded to the stimuli by pressing one of the two keys: A for red or circle, and L for green or triangle. To maintain the age-appropriateness, the number of blocks and trials were modified according to Antoniou et al. [63]. Children started with two training blocks of 32 trials each, where they were informed which characteristic to focus on, before each block (i.e., *sort the target stimuli by shape* or *sort the target stimuli by colour*) and they received feedback on incorrect trials (i.e., *Incorrect* appears in the middle of the screen). This was followed by two mixed-condition testing blocks of 32 trials each, where the children were asked to alternate between colour and shape depending on the letter cue (i.e., C = Colour or S = Shape) preceding each trial on the top of the screen. The letter cue (i.e., C or S) was presented alone for 500 ms, then remained on screen with the target stimulus until response. After the response, a blank inter-trial interval of 600 ms preceded the next trial. No error feedback was given in the test blocks which contained an equal number of shift and repeat trials. Shift trials occurred when children had to apply a different sorting rule than on the previous trial (e.g., shape followed by colour) and repeat trials were when the same sorting rule was applied as the previous trial (e.g., shape followed by shape). In accord with Eich et al. [64], shifting effectiveness was operationalized as the percentage of correct shift trials: number of correct shifts/total shift trials x 100. Shifting efficiency was operationalized as the percentage of correct shift trials divided by mean RT for correct shift trials. Higher effectiveness values indicate more accurate shifting, while higher efficiency values indicate faster and more accurate shifting performance. As for inhibitory efficiency, shifting efficiency was also multiplied by 1,000 to improve interpretability.

**Updating.** An *N*-back task (As some participants found the *3*-back condition of the *n*-back task challenging, sensitivity analyses were conducted excluding *3*-back trials. These analyses yielded comparable patterns of results for updating effectiveness and efficiency, so all reported analyses are based on the full set of *n*-back trials.) [65] was used to index updating performance. The task required children to monitor letters presented one at a time, in blocks of increasing difficulty (i.e., *n*) and indicate when a letter had been seen in the previous trial (*1*-back), after 1 intervening trial (*2*-back), or after 2 intervening trials (*3*-back). Difficulty increased with the number of intervening trials. Children indicated the same (Yes-trial) or different (No-trial) with a keyboard button press based on the letter *1*-, *2*-, or *3*-back from this letter. Following Pelegrina et al. [66], each *n*-back block (e.g., *1*-back) comprised an instruction, an example including a sequence of four letters with the demonstration of a correct response, a practice block containing 20 trials, and two testing blocks containing 20 trials for each level of difficulty. Testing blocks contained 30% Yes-trials. Trials begun with an empty black screen for 500 ms, followed by presentation of a single letter for 500 ms, then a blank 3000 ms inter-stimulus interval. The first three trials in each block were always No-trials. Following Wong et al. [67], updating effectiveness was captured by discrimination sensitivity (*d*’): *z-*score for correct Yes-trials minus *z-*score No-trial errors [61]. Updating efficiency was calculated as *d’* divided by mean RT for correct Yes-trials. Higher effectiveness values indicate better discrimination between Yes- and No-trials, while higher efficiency values indicate faster and more accurate updating performance. In line with the other efficiency measures, updating efficiency was multiplied by 1,000 to enhance interpretability.

### Math achievement measures

Three subtests from the Woodcock-Johnson IV Tests of Achievement [68] were used to assess math achievement, namely, Applied Problems, Calculation, and Math Fact Fluency. To avoid measurement error due to the practice effects, Form C was used at pre-training, Form A was used at post-training, Form B was used at 1-month follow-up, and Form C was used again at 3-month follow-up. Form C includes Australian norms through a localized standardization process. To improve cultural and linguistic relevance of Form A and Form B, we made minor adaptations to the wording of some test items (e.g., ‘*gas*’ was changed to ‘*petrol*’, ‘*miles*’ to ‘*kilometres*’, ‘*dimes*’ and ‘*nickels*’ to ‘*cents*’ etc) to better reflect Australian English usage and measurement systems. Administration and scoring were consistent with the authors’ manual. Math achievement was indexed using the Broad Mathematics cluster score; calculated by averaging the child’s W-scores for each of the three subtests, i.e., Applied Problems, Calculation, and Math Fact Fluency [68].

### Training tasks

Both training tasks used the same game narrative. Children were told they were playing the role of an explorer who accidentally awakened a dragon in a cave. To escape from the dragon, the explorer must travel across the world over 15 days. Children were instructed to collect as much treasure (gold and gems) as possible to pay for their travel. The journey began in Australia on the first day and continued to a different country each day (e.g., France, China, Italy, Vietnam etc). A token-based reward system was implemented whereby children gained treasure if they responded correctly, and lost treasure for responding incorrectly.

**Fig 1:**
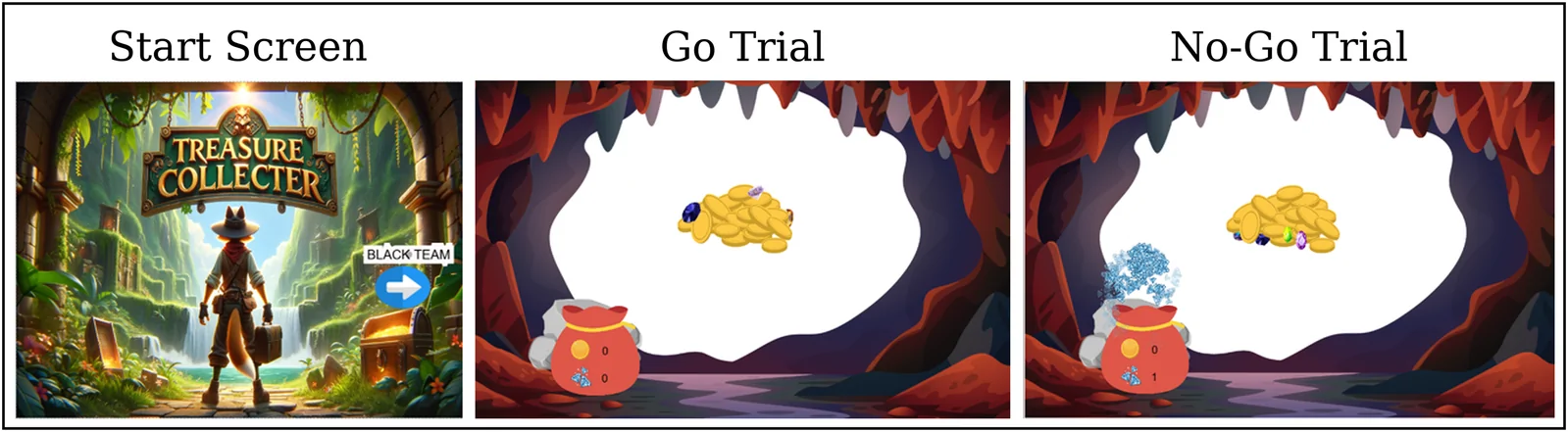
Author-developed Inhibitory Control Training Task (screenshots). The graphical assets used within the inhibitory control training task shown in this figure were generated using ChatGPT (OpenAI) and subsequently integrated into the game by the authors.

**Inhibitory control training task.** The inhibitory control training group trained using a game based on an adaptive Go/No-Go (aGo/No-Go) task (see Fig 1). Children were presented with a continuous stream of blended stimuli: piles of gold either with or without a green gem. They were asked to respond as quickly and accurately as possible, based on instructions, e.g., *When you see a PILE OF GOLD, hit the SPACEBAR to add it to your treasure* (Go) *but if you see a GREEN gem hidden in the pile of gold, this is dangerous, and you must not hit the SPACEBAR, just do nothing* (No-Go). This reversed the dominant real-world association of the colour green with Go (e.g., traffic signals) requiring participants to inhibit an automatic response. The task was purposefully designed to challenge this habitual colour-response link and activate inhibitory control. To increase cognitive demand, in No-Go stimuli, green gems varied in shape and were accompanied by 2–5 similar-shaped but differently coloured gems. Go stimuli also included 2–5 same-shaped but differently coloured gems to increase visual complexity. Children began by completing 10 practice trials to ensure they understood the rules and structure. The main training task followed, comprising six blocks of 50 trials each (300 trials total), with 50% Go and 50% No-Go stimuli [39,69]. Each stimulus was displayed for 1,000 ms. Inter-stimulus intervals started at 1,000 ms and

were adjusted across blocks based on the accuracy rate of the preceding block, specifically, the inter-stimulus intervals increased by 200 ms if accuracy was below 75%, decreased by 200 ms if accuracy exceeded 90%, and remained unchanged if accuracy was between 75–90% [70,71]. Children were instructed to take a 10-second break between each block. Following other studies who have examined performance on an inhibitory control training task across sessions, we operationalized performance as the false alarm rate (number of false alarms or No-Go errors divided by the number of trials) across all blocks in the training session [72]. As such, lower false alarm rates indicated improved inhibitory control, as they reflect more successful withholding of prepotent responses.

**Fig 2:**
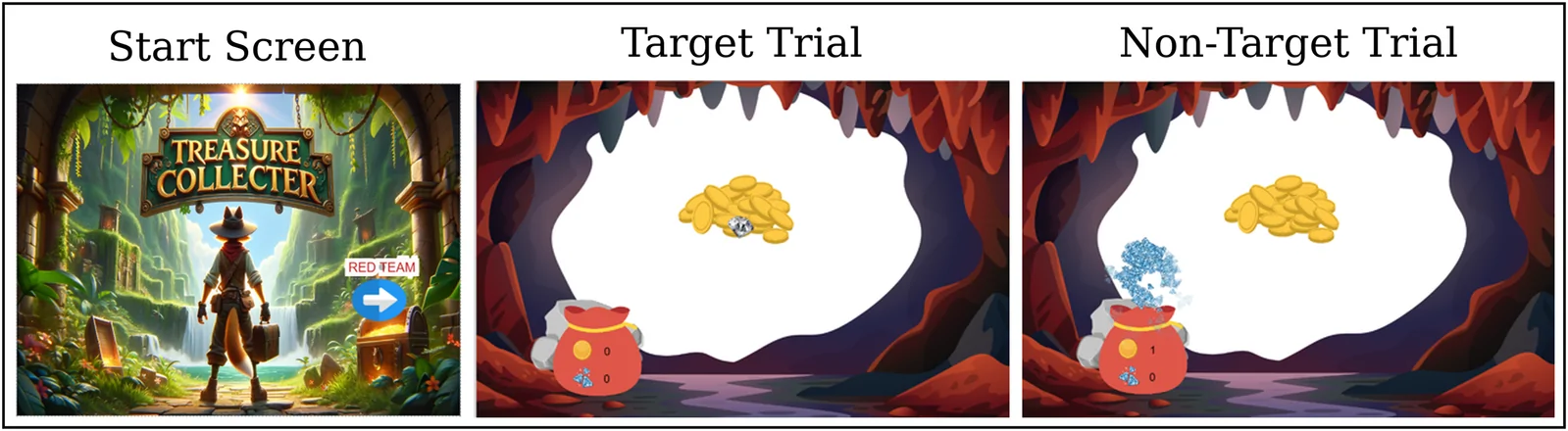
Author-developed Visual Identification Training Task (screenshots). The graphical assets used within the visual identification training task shown in this figure were generated using ChatGPT (OpenAI) and subsequently integrated into the game by the authors.

**Visual identification training task.** The active control group trained using a game based on a low-load visual identification task designed to match the inhibitory control training task on features such as block and trial count, stimuli ratio, stimuli duration, inter-stimulus intervals, and difficulty adaptation, yet without the requirement to withhold (inhibit) a dominant response (see Fig 2). Children were presented with a continuous stream of stimuli of piles of gold that either contained a diamond (target) or not (non-target). Unlike the inhibitory control training task, children were not required to suppress or inhibit responses. Instead, they were instructed to press the spacebar as quickly as possible whenever a target stimulus appeared. Thus, the task was designed to engage visual attention and motor responding without systematically taxing inhibitory control processes theoretically implicated in anxiety and emotional regulation. This approach is consistent with prior cognitive control training studies that have used low-load speeded-response tasks as active control conditions (e.g.[15,18],) and was conceptually similar to the control task employed by Ganesan et al. [42]. By matching the intervention on general engagement, expectancy, contact time, and game exposure while omitting inhibitory demands, the active control condition strengthened causal inference regarding whether any observed effects could be specifically attributed to inhibitory control training rather than non-specific intervention effects. We operationalized performance as the non-target error rate (number of non-target errors divided by the number of trials) across all blocks in the training session.

### Procedure

Data were collected one-on-one with a researcher, in a quiet room adjacent to the child’s regular classroom. To avoid fatigue, data at pre-, post-training, 1-month, and 3-month follow-up were collected over multiple sessions, lasting no longer than 40 minutes each. In the first pre-training session, children completed the Math Learning Anxiety subtest from the Modified Abbreviated Math Anxiety Scale followed by the math achievement measures from the Woodcock-Johnson IV Tests of Achievement., namely, the Applied Problems, Calculation, and Math Fact Fluency subtests. In the second session, children completed the Spence Children’s Anxiety Scale, the Penn State Worry Questionnaire for Children, the Low Mood subscale from the Revised Children’s Anxiety and Depression Scale, and Rumination subscale from the Child Response Style Questionnaire, followed by three cognitive measures, specifically, the Go/No Go task (inhibitory control), Colour Shape-Shifting task (shifting) and the *N*-back task (updating). To control for order effects, the cognitive tasks were administered using a Latin square design. In the next session, children were introduced to the cognitive training. They were told there would be two ‘training games’ and they must only play the game they were allocated. Researchers referred to the two groups as either the ‘Red Group’ or the ‘Black Group’ yet neither the children, nor their guardians and teachers were aware of the differences between the games/tasks/groups. Children were asked to complete 15 sessions of training, each lasting approximately 10 minutes (dependent on their performance) on school days under teacher supervision. Immediately post-training, and again at 1- and 3-month follow-up the same emotional, academic and cognitive measures were repeated as at baseline. To enhance engagement and adherence, teachers displayed a chart in the classroom, with children colouring a grid after each completed training session to provide a visual record of progress. On completion of the study, children were thanked and remunerated with a $40 gift card for their participation. To incentivise recruitment and compensate teachers for their time supervising training, schools were provided with funds equivalent to the cost of one relief teacher (also known as a supply or substitute teacher) per class of 20 participating children for each data collection point.

### Statistical analysis

ITT analyses were selected as the primary analytic approach because they preserve the benefits of randomization, reduce bias associated with non-adherence and attrition, and provide a conservative estimate of intervention effects under real-world implementation conditions [73]. This was particularly relevant in the present trial, as the intervention was delivered to a universal sample in school settings, where variation in training completion was expected.

All analyses were conducted in R (version 4.5.2; see supplemental materials for R-package version info in S1 File). To account for the repeated measures structure of the data, we used linear mixed models (LMMs) (We deviated from the pre-registered analysis plan by using linear mixed models instead of mixed ANOVAs. This approach is better suited for RCT data with missing observations because it uses all available data without listwise deletion and accounts for the hierarchical structure of the repeated measures. In line with this, recent cognitive control training studies in adults have relied on similar analytical approaches (e.g., Vander Zwalmen et al., 2025 [74]).) to examine the effects of inhibitory control training on cognitive performance (inhibition, shifting and updating effectiveness and efficiency), emotional outcomes, and math achievement. This method accommodates missing observations and individual differences in the number of completed sessions. Separate LMMs were fitted for each outcome variable using restricted maximum likelihood estimation. For the categorical predictors Group and Time, dummy coding was used, with the active control group and the baseline (pre-training) timepoint as the reference levels. Accordingly, intervention effects were evaluated based on differential change over time relative to baseline rather than post-intervention group differences alone.

At Level 1, the outcome for individual *j* at Time *t* was modelled as a function of Group (factor with two levels: inhibitory control training vs active control) and Time (factor with four levels: pre-training, post-training, 1-month follow-up and 3-month follow-up). We also included a Time × Group interaction to test whether changes in each outcome over time differed between the two training conditions.


$$\begin{array}{c} {Outcome}_{tj}=\;\beta_{\text{0}j}+\;\beta_{\text{1}j}{Group}_{j}+\;\beta_{\text{2}j}{Time}_{tj}+\;\beta_{\text{3}j}{Group}_{j}\times {Time}_{tj}+e_{tj} \end{array}$$

At Level 2, for each outcome we compared a model including only a random intercept for participant *j* (β_*0j*_) with a more complex model including both a random intercept and a random slope for Time (β_*1j*_). The random slope used a numeric coding of Time to limit the number of parameters and ensure stable model estimation. The two models were compared using a likelihood-ratio test to determine whether allowing a random slope for Time improved model fit. When the more complex model fit significantly better, it was retained; otherwise, the more parsimonious model was used.


$$\begin{array}{c} \beta_{\text{0}j}=\;\gamma_{\text{00}}+\;u_{\text{0}j}\;(\text{random intercept}) \end{array}$$

$$\begin{array}{c} \beta_{\text{2}j}=\;\gamma_{\text{20}}+\;u_{\text{2}j}\;(\text{random slope}) \end{array}$$

Here, β_0*j*_ is the intercept for participant *j*, and β_*2j*_ is the Time slope for participant *j*. γ_00_ and γ_20_ represent the average intercept and average Time effect across participants, whereas *u*_*0j*_ and *u*_*2j*_ reflect each participant’s deviation from these averages.

To facilitate the interpretation of the magnitude and practical significance of the observed effects, standardized regression coefficients (*β*_std_) were calculated for each LMM using the *effectsize* R package [75]. These standardized coefficients were reported as effect size estimates and interpreted as follows: *β*_std_ values below 0.2 were considered small, values between 0.2 and 0.5 medium, and values above 0.5 large [76]. In addition, we visually assessed model assumptions of linearity, homoscedasticity and normality of residuals and random effects using the *performance* package [77]. No major violations of model assumptions were observed. Across all analyses, significant Group × Time interaction effects were followed up with simple slope analyses to facilitate interpretation, using the *reghelper* package [78]. In line with the protocol, we applied Bonferroni corrections to follow-up tests to control for type 1 error, and statistical significance was set at α = .05.

### Additional analyses

In addition to the ITT-analyses, we conducted the same LMM analyses using a per-protocol sample, which included participants who completed eleven or more training sessions (*N* = 96). A descriptive table with the mean outcome per group per timepoint for this population can be found in the supplementary materials (Supplemental Table 2 in S1 File).

## Results

### Data reduction

**Fig 3:**
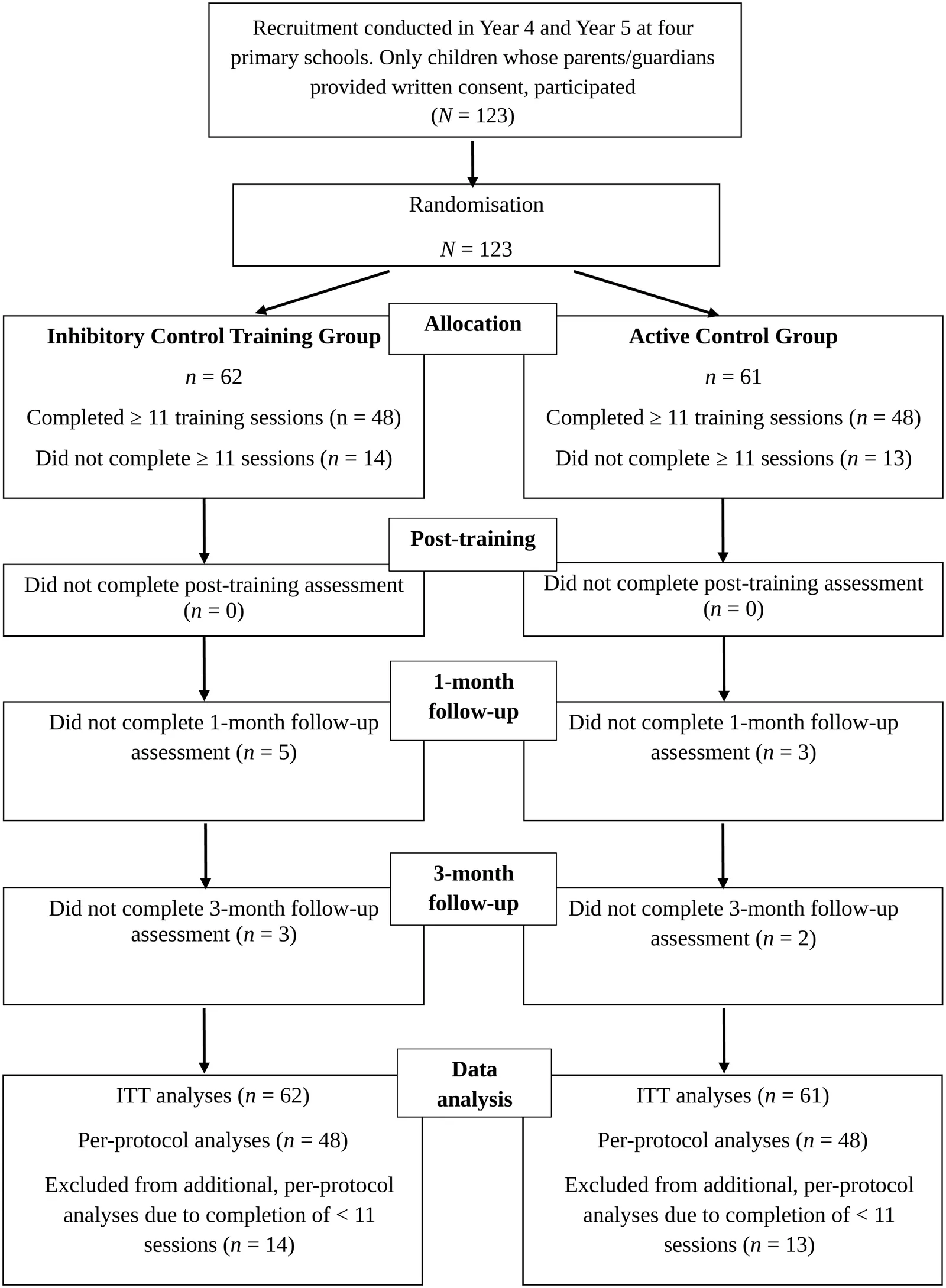
CONSORT flow diagram.

Prior to conducting the analyses, data cleaning was undertaken to screen for missing values and response inconsistencies. Cleaning the cognitive measures data was done at the individual level. To avoid non-representative responses, RTs < 200 ms were considered anticipatory and removed, and RTs ± 3SD from each participant’s mean score were also removed (< 1% of trials). The training data were inspected for adherence to the assigned training task at the individual session level. All participants who were randomized were included in the ITT analyses (*N* = 123). Only participants who completed eleven or more training sessions (*n* = 96) were included in the per-protocol analyses [25,46]. The CONSORT flow diagram for the study is shown in Fig 3.

To examine the possibility of attrition-related bias, baseline characteristics were compared between participants who completed at least eleven training sessions (‘completers’) and those who completed fewer than eleven sessions (‘non-completers’). The results indicated that non-completers reported significantly higher baseline levels of worry than completers (see Table 2). No significant differences were observed for the demographic, cognitive, academic, or other emotional variables. This pattern suggests that children with higher initial worry may have been more likely to discontinue the intervention. As noted above, the ITT analyses minimizes bias associated with attrition and non-adherence by including all randomized participants in linear mixed-effects models. Accordingly, the per-protocol analyses should be interpreted with caution due to potential bias arising from differential attrition.

**Table 2:** Baseline Characteristics of Completers vs Non-Completers.

	Completers*n* = 96	Non-Completers*n* = 27		
	*Mean/n (SD/%)*	*Mean/n (SD/%)*	*t/χ2*	*p*
**Demographic**				
Age in years	9.74 (0.81)	9.93 (0.73)	1.14	.259
Sex			< .001	1.000
Male	50 (52.08%)	14 (51.85%)		
Female	46 (47.92%)	13 (48.15%)		
**Emotional measures**				
Anxiety	27.38 (19.66)	37.56 (25.00)	1.95	.059
Worry	15.68 (8.33)	21.04 (10.19)	2.50	.017
Depression	9.18 (6.74)	11.44 (7.67)	1.39	.172
Rumination	13.36 (9.95)	17.74 (10.18)	1.98	.054
Math Anxiety	9.39 (4.34)	9.59 (3.98)	0.23	.820
**Cognitive measures**				
Inhibitory Effectiveness	2.64 (0.82)	2.41 (0.71)	−1.47	.147
Inhibitory Efficiency	4.41 (1.53)	4.42 (1.55)	0.06	.955
Shifting Effectiveness	64.62 (19.36)	61.13 (21.56)	−0.75	.459
Shifting Efficiency	49.41 (24.72)	53.35 (25.30)	0.71	.484
Updating Effectiveness	1.11 (0.69)	1.12 (0.80)	0.11	.916
Updating Efficiency	1.31 (0.86)	1.48 (1.18)	0.68	.504
**Math achievement measure**				
Math Achievement	489.73 (15.44)	489.70 (13.77)	−0.01	.994

*Note.* Mean (*SD*). Efficiency measures are multiplied by 1,000 to improve interpretability.

### Performance on training tasks

As a manipulation check, we conducted LMM analyses on the training task data. The models used Maximum Likelihood estimation and included a fixed effect of training day, with random intercepts for participants.

### Inhibitory control training task

To determine whether performance on the inhibitory control training task improved over the training sessions, we conducted an LMM analysis with false alarm rate (i.e., No-Go errors/number of trials) as the dependent variable [72]. Lower scores indicated better inhibitory control. Results showed a significant improvement in performance across training, *b* = −0.0009, 95% Confidence interval (CI) = [−0.0016, −0.0003], *p* = .006 suggesting that children made fewer false alarms (No-Go errors) as training progressed, indicating enhanced performance over time.

### Visual identification task

To determine whether performance changed across training, we conducted an LMM analysis with non-target error rate (i.e., non-target errors/number of trials) as the dependent variable. Lower scores indicated fewer non-target errors and better performance. Results showed no evidence of systematic change in task performance across training, *b* = 0.00020, 95% CI = [−0.00022, 0.00061], *p* = .360. Given that the visual identification task was deployed to control for study-related effects, such as contact with researchers and engagement with playing a daily game, these results support the task as an appropriate comparison condition.

### Training effects on cognitive performance

To test H1, we conducted separate LMMs in the ITT sample for inhibition, shifting, and updating effectiveness and efficiency. Likelihood-ratio tests indicated that a model including a random intercept only fit the data better than a more complex model including a random intercept and a random slope for Time for inhibitory efficiency. For the other five outcomes, a more complex model including a random intercept and a random slope provided a better fit and was used in the analyses. Full model results are shown in Table 3.

**Table 3:** LMM results for cognitive performance measures – ITT sample.

Outcome	Predictors	*ß*	*SE*	*df*	*t*	*p*	*ß_std_* [95% CI]
Inhibitory	Intercept	2.60	0.11	174.04	24.61	< .001	0.25 [0.02, 0.48]
Effectiveness	Group	-0.01	0.15	174.03	-0.10	.923	-0.02 [-0.34, 0.31]
	Timepoint 2	-0.16	0.11	297.09	-1.40	.163	-0.18 [-0.43, 0.07]
	Timepoint 3	-0.04	0.12	340.20	-0.35	.729	-0.05 [-0.31, 0.22]
	Timepoint 4	-0.22	0.13	142.31	-1.76	.081	-0.24 [-0.52, 0.03]
	Group × Timepoint 2	-0.23	0.16	297.07	-1.45	.148	-0.26 [-0.61, 0.09]
	Group × Timepoint 3	-0.44	0.17	339.42	-2.62	.009	-0.49 [-0.86, -0.12]
	Group × Timepoint 4	-0.24	0.18	141.73	-1.34	.181	-0.26 [-0.65, 0.12]
Inhibitory	Intercept	4.48	0.21	255.93	21.10	< .001	0.32 [0.07, 0.57]
Efficiency	Group	-0.12	0.30	255.91	-0.40	.688	-0.07 [-0.42, 0.28]
	Timepoint 2	-0.28	0.20	355.38	-1.39	.165	-0.17 [-0.40, 0.07]
	Timepoint 3	-0.35	0.21	356.63	-1.73	.085	-0.21 [-0.45, 0.03]
	Timepoint 4	-0.53	0.20	355.79	-2.60	.010	-0.31 [-0.55, -0.08]
	Group × Timepoint 2	-0.44	0.28	355.38	-1.54	.126	-0.26 [-0.59, 0.07]
	Group × Timepoint 3	-0.62	0.29	356.41	-2.14	.033	-0.37 [-0.70, -0.03]
	Group × Timepoint 4	-0.42	0.29	355.58	-1.49	.137	-0.25 [-0.58, 0.08]
Shifting	Intercept	64.42	2.74	160.55	23.53	< .001	0.06 [-0.17, 0.29]
Effectiveness	Group	-1.32	3.85	159.63	-0.34	.731	-0.06 [-0.38, 0.27]
	Timepoint 2	1.61	2.60	301.49	0.62	.536	0.07 [-0.15, 0.29]
	Timepoint 3	2.44	2.77	328.19	0.88	.380	0.10 [-0.13, 0.34]
	Timepoint 4	4.64	2.96	135.95	1.57	.119	0.20 [-0.05, 0.45]
	Group × Timepoint 2	-7.24	3.65	300.15	-1.99	.048	-0.31 [-0.62, -0.003]
	Group × Timepoint 3	-4.40	3.89	327.76	-1.13	.260	-0.19 [-0.51, 0.14]
	Group × Timepoint 4	-10.96	4.15	134.78	-2.64	.009	-0.47 [-0.82, -0.12]
Shifting	Intercept	48.36	3.45	160.88	14.00	< .001	-0.19 [-0.44, 0.07]
Efficiency	Group	4.28	4.85	159.96	0.88	.379	0.16 [-0.20, 0.52]
	Timepoint 2	9.65	3.47	305.12	2.78	.006	0.36 [0.11, 0.62]
	Timepoint 3	7.86	3.80	315.01	2.07	.040	0.29 [0.01, 0.57]
	Timepoint 4	8.22	4.19	135.00	1.96	.052	0.31 [0.001, 0.62]
	Group × Timepoint 2	-5.48	4.88	303.90	-1.12	.262	-0.21 [-0.56, 0.15]
	Group × Timepoint 3	-9.85	5.34	314.55	-1.85	.066	-0.37 [-0.76, 0.02]
	Group × Timepoint 4	-11.65	5.88	133.80	-1.98	.0496	-0.44 [-0.87, -0.004]
Updating	Intercept	1.08	0.10	155.03	11.27	< .001	0.12 [-0.12, 0.36]
Effectiveness	Group	0.07	0.13	154.12	0.56	.580	0.10 [-0.24, 0.44]
	Timepoint 2	0.02	0.09	304.22	0.24	.809	0.03 [-0.19, 0.24]
	Timepoint 3	-0.06	0.09	328.36	-0.61	.542	-0.07 [-0.31, 0.16]
	Timepoint 4	-0.04	0.10	142.04	-0.46	.649	-0.06 [-0.31, 0.19]
	Group × Timepoint 2	-0.32	0.12	302.99	-2.66	.008	-0.41 [-0.72, -0.11]
	Group × Timepoint 3	-0.27	0.13	327.21	-2.07	.039	-0.34 [-0.67, -0.02]
	Group × Timepoint 4	-0.28	0.14	140.83	-1.99	.048	-0.35 [-0.70, -0.005]
Updating	Intercept	1.28	0.13	155.16	9.67	< .001	0.06 [-0.20, 0.31]
Efficiency	Group	0.13	0.19	153.37	0.70	.487	0.13 [-0.23, 0.48]
	Timepoint 2	0.11	0.12	301.15	0.95	.345	0.11 [-0.12, 0.34]
	Timepoint 3	-0.07	0.13	313.48	-0.58	.566	-0.07 [-0.32, 0.17]
	Tim point 4	0.05	0.14	145.91	0.34	.733	0.05 [-0.22, 0.32]
	Group × Timepoint 2	-0.39	0.17	298.70	-2.29	.023	-0.37 [-0.69, -0.05]
	Group × Timepoint 3	-0.35	0.18	313.17	-1.92	.056	-0.33 [-0.68, 0.01]
	Group × Timepoint 4	-0.49	0.20	140.36	-2.47	.015	-0.47, [-0.85, -0.10]

Timepoint 1 = Pre-training; Timepoint 2 = Post-training, Timepoint 3 = 1-month follow-up, Timepoint 4 = 3-month follow-up. CI = Confidence interval.

Across all executive function measures, there were no significant main effects of Group at baseline, indicating that participants in the inhibitory control training and active control groups started at comparable levels. However, the LMMs did reveal significant Group × Time interactions for all executive function outcomes, suggesting differential change over time between the inhibitory control training group and the active control group.

**Fig 4:**
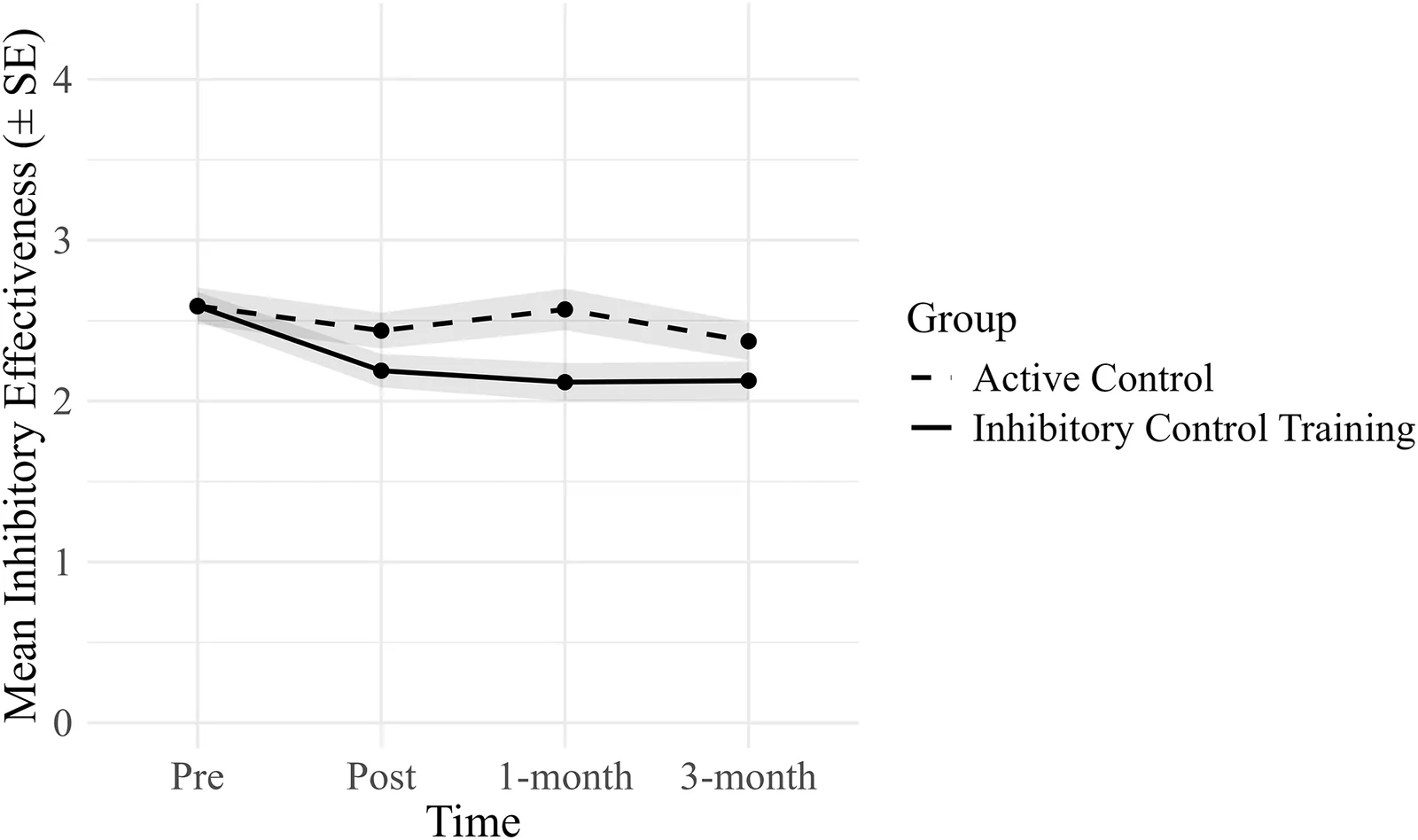
Mean Inhibitory Effectiveness per Training Group Across Time – ITT. *SE* = Standard Error.

For inhibitory effectiveness, the LMM revealed a significant Group × Time interaction at 1-month follow-up (*β* = −0.44, *SE* = 0.17, *t(339)* = −2.62, *p* = .009, *ß*_*std*_ = 0.49, 95% CI = [−0.86, −0.12]), indicating that changes in inhibitory effectiveness across time differed between the inhibitory control training and active control groups. Fig 4 shows the mean inhibitory effectiveness across time for each group. Follow-up simple-slope analyses helped clarify the direction of these interactions. Within the inhibitory control training group, inhibitory effectiveness decreased significantly from pre-training to 1-month follow-up (*β* = −0.49, *SE* = 0.12, *t(339)* = −4.08, *p* < .001). In contrast, the active control group showed no significant changes from pre-training to the 1-month follow-up (*β* = −0.04, *SE* = 0.12, *t(340)* = −0.35, *p* = .729). After applying a Bonferroni correction for multiple testing, the effect for the inhibitory control group remained significant.

**Fig 5:**
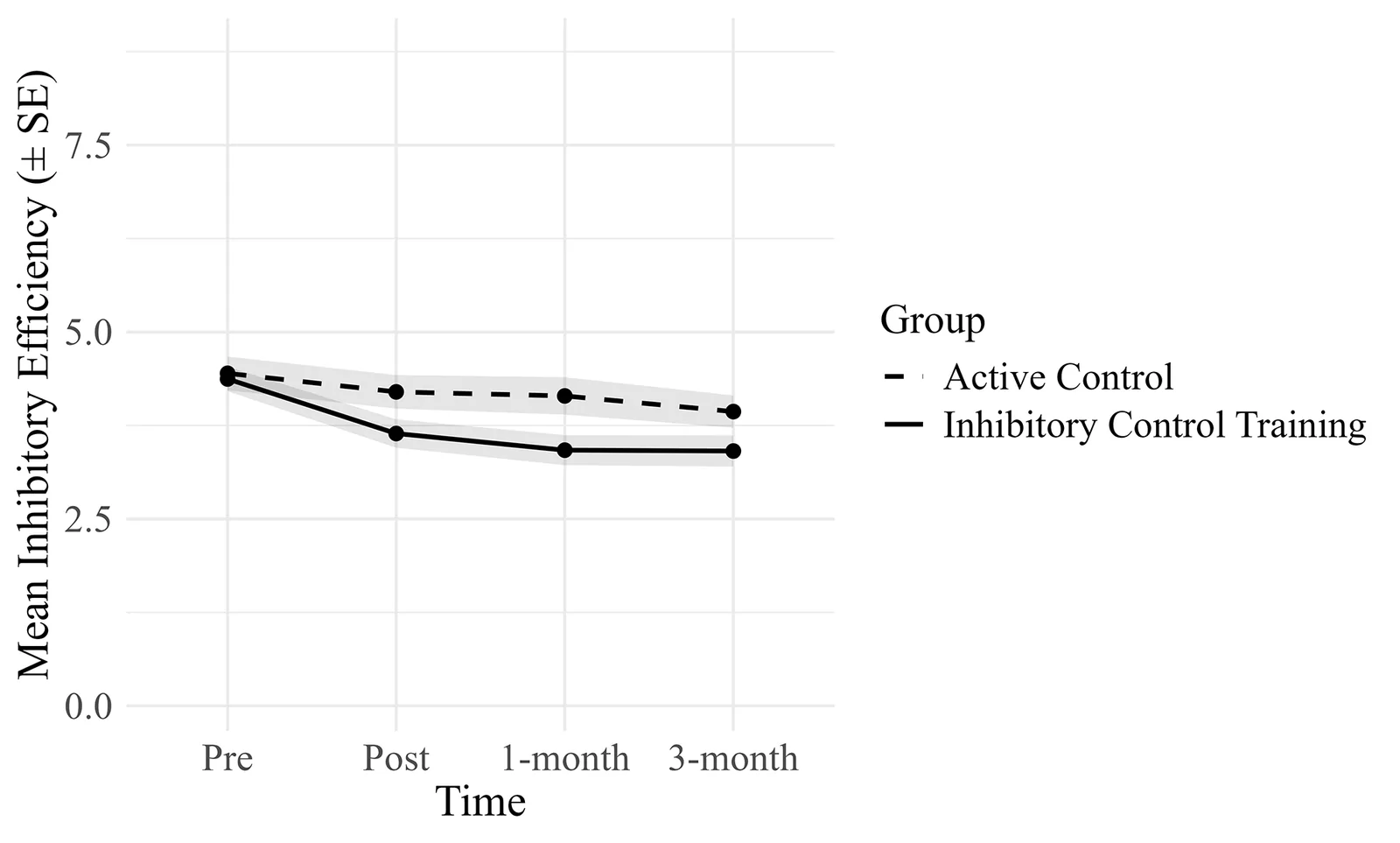
Mean Inhibitory Efficiency per Training Group Across Time – ITT. *SE* = Standard Error.

Regarding inhibitory efficiency, the Group × Time interaction was also significant at the 1-month follow up (*β* = −0.62, *SE* = 0.29, *t(356)* = −2.14, *p* = .033, *β*_*std*_ = 0.-37, 95% CI = [−0.70, −0.03]) suggesting that changes in this measure over time depended on the training group (Fig 5). Follow-up simple slope analyses revealed that within the inhibitory control training group, inhibitory efficiency decreased significantly from pre-training to the 1-month follow-up (*β* = −0.97, *SE* = 0.20, *t(356)* = −4.80, *p* < .001), whereas the active control group showed no significant change (*β* = −0.35, *SE* = 0.21, *t(357)* = −1.73, *p* = .085). The observed change in inhibitory efficiency from baseline to the 1-month follow-up remained significant after applying Bonferroni correction.

**Fig 6:**
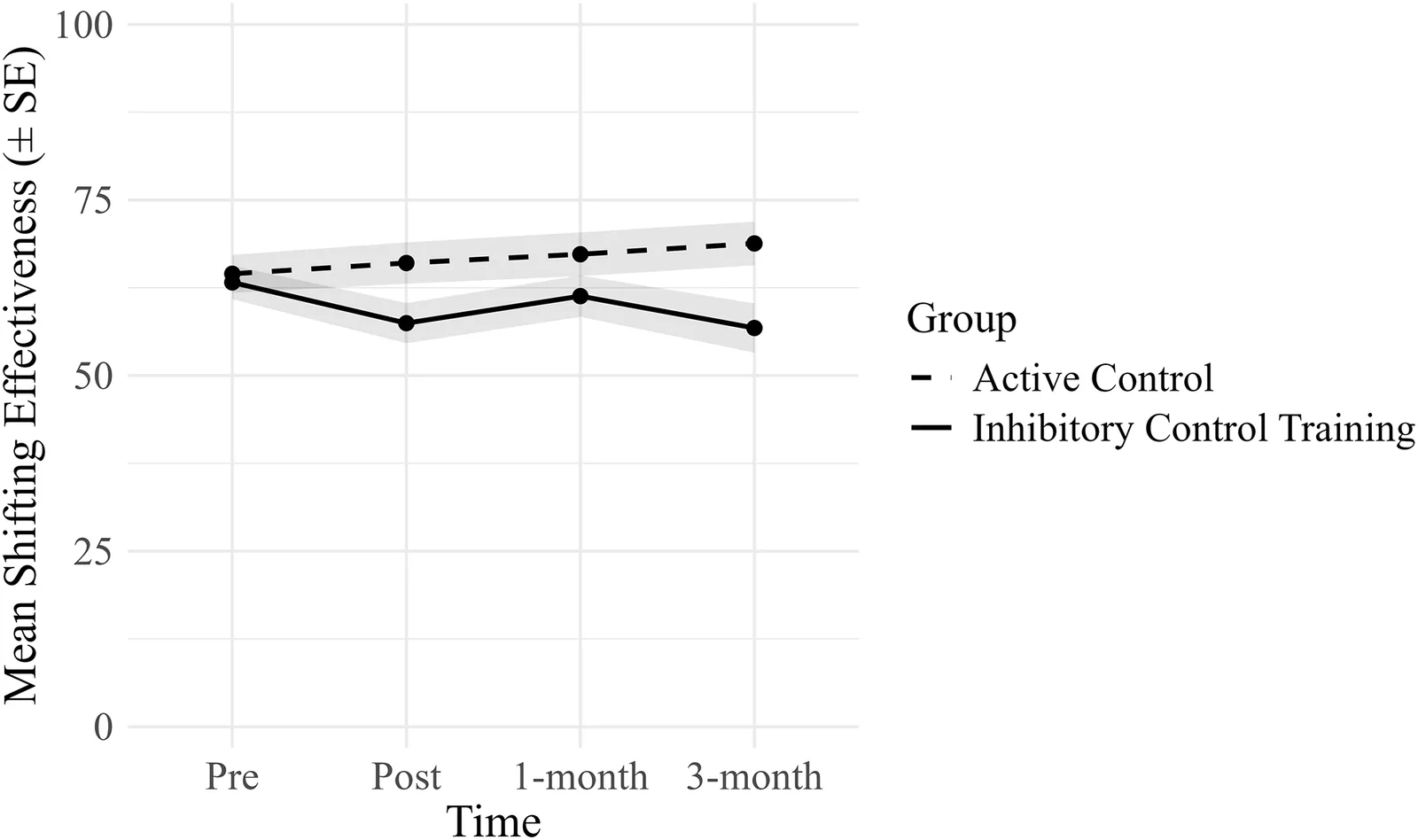
Mean Shifting Effectiveness per Training Group Across Time – ITT. *SE* = Standard Error.

Next, for shifting effectiveness, significant Group × Time interactions were found at post-training (*β* = −7.24, *SE* = 3.65, *t(300)* = −1.99, *p* = .048, *β*_*std*_  = −0.31, 95% CI = [−0.62, −0.003]) and at the 3-month follow-up (*β* = −10.96, *SE* = 4.15, *t(135)* = −2.64, *p* = .009, *β*_*std*_  = −0.47, 95% CI = [−0.82, −0.12]), indicating that changes in shifting effectiveness over time differed between the training groups (Fig 6). Simple slopes showed that within the inhibitory control training group, shifting effectiveness decreased significantly from pre- to post-training (*β* = −5.63, *SE* = 2.56, *t(299)* = −2.20, *p* = .029) and from pre-training to the 3-month follow-up (*β* = −6.32, *SE* = 2.91, *t(134)* = −2.17, *p* = .031). No significant changes were observed in the active control group at either time point (post-training: *β* = 1.61, *SE* = 2.60, *t(301)* = 0.62, *p* = .536; 3-month follow-up: *β* = 4.64, *SE* = 2.96, *t(136)* = 1.57, *p* = .120). However, after applying Bonferroni correction, none of the simple slope effects remained statistically significant.

**Fig 7:**
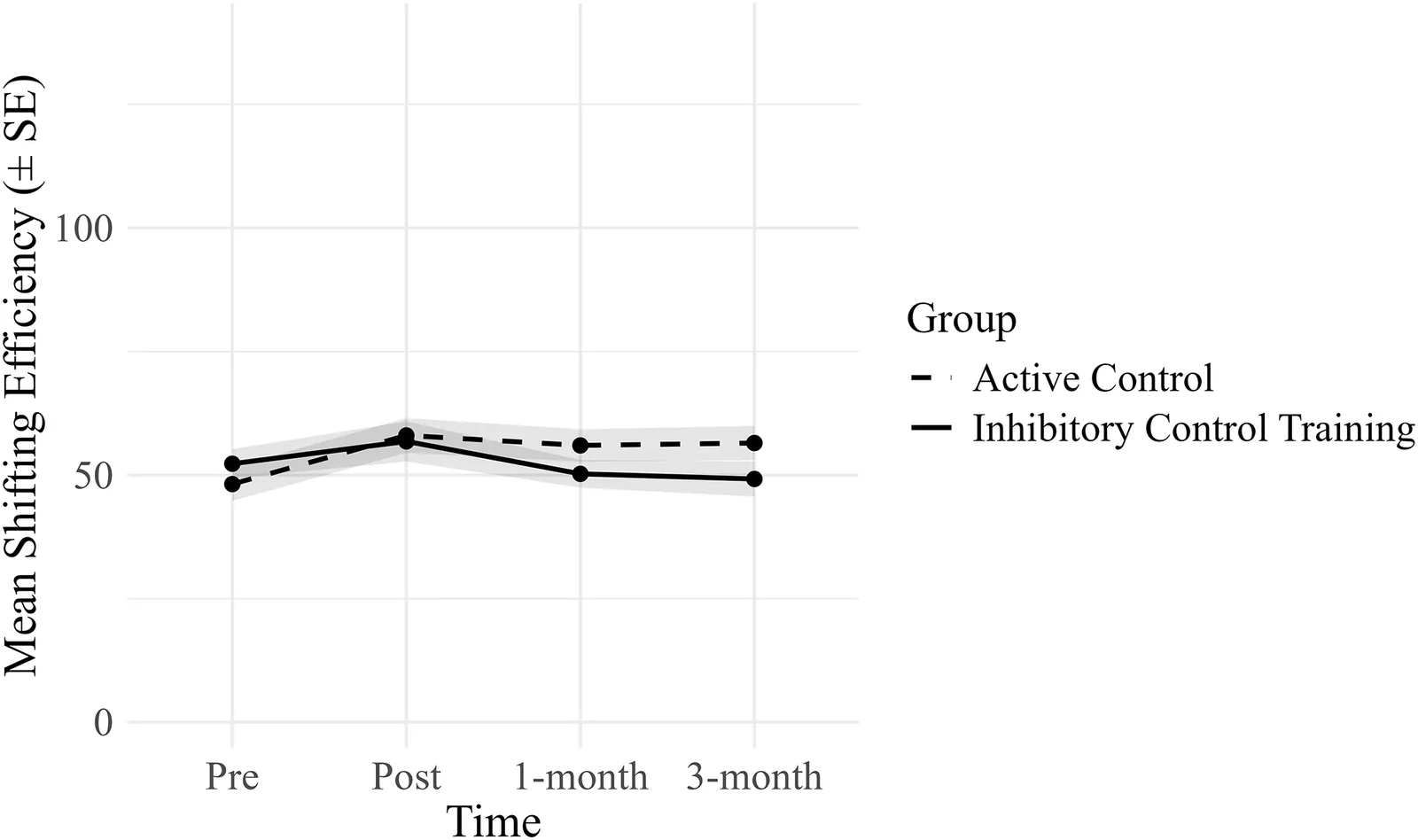
Mean Shifting Efficiency per Training Group Across Time – ITT. *SE* = Standard Error.

Regarding shifting efficiency, a significant Group × Time interaction at the 3-month follow-up (*β* = −11.65, *SE* = 5.88, *t(138)* = −1.98, *p* = .0496, *β*_*std*_  = −0.44, 95% CI = [−0.87, −0.004]) suggested differential changes in shifting efficiency across time between both groups (Fig 7). Follow-up simple slopes however, revealed no significant changes from pre-training in the inhibitory control group (*β* = −3.43, *SE* = 4.12, *t(133)* = −0.83, *p* = .407), nor in the active control group (*β* = 8.22, *SE* = 4.19, *t(135)* = 1.96, *p* = .052). This pattern remained the same after applying Bonferroni correction.

**Fig 8:**
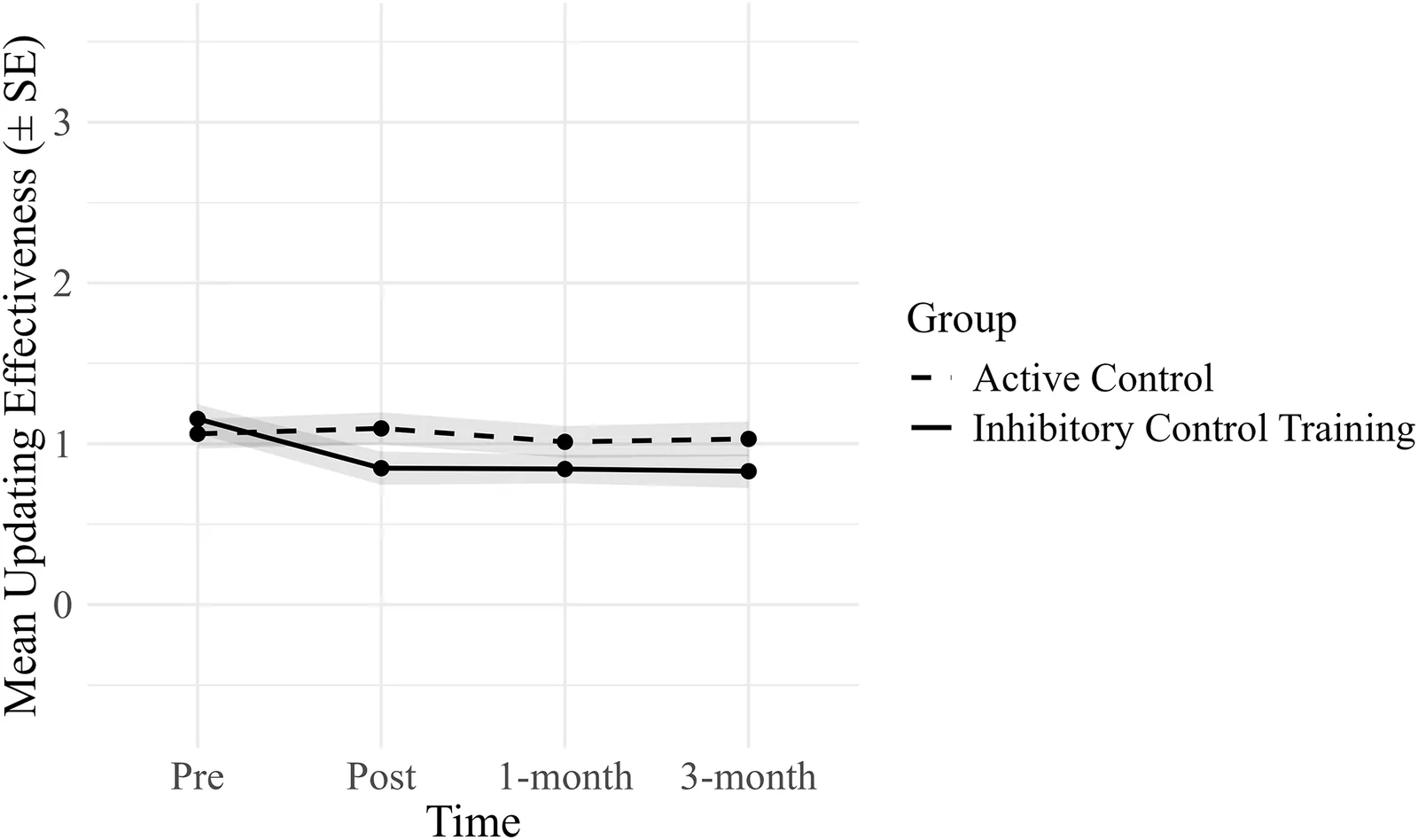
Mean Updating Effectiveness per Training Group Across Time – ITT. *SE* = Standard Error.

The LMM for updating effectiveness revealed significant Group × Time interactions at all three time points (post-training: *β* = −0.32, *SE* = 0.12, *t(303)* = −2.66, *p* = .008, *β*_*std*_  = −0.41, 95% CI = [−0.72, −0.11]; 1-month follow-up: *β* = −0.27, *SE* = 0.13, *t(327)* = −2.07, *p* = .039, *β*_*std*_  = −0.34, 95% CI = [−0.67, −0.02]; 3-month follow-up: *β* = −0.28, *SE* = 0.14, *t(141)* = −1.99, *p* = .048, *β*_*std*_  = −0.35, 95% CI = [−0.70, −0.005]), indicating that temporal changes in updating effectiveness depended on the training group (see Fig 8). Simple slopes revealed significant decreases in the outcome from baseline at each of these three time points for the inhibitory control training group (post-training: *β* = −0.30, *SE* = 0.09, *t(302)* = −3.53, *p* < .001; 1-month follow-up: *β* = −0.32, *SE* = 0.09, *t(326)* = −3.57, *p* < .001; 3-month follow-up: *β* = −0.32, *SE* = 0.10, *t(140)* = −3.31, *p* = .001), whereas no significant changes from pre-training at either time point were observed in the active control condition (post-training: *β* = 0.02, *SE* = 0.09, *t(304)* = 0.24, *p* = .809; 1-month follow-up: *β* = −0.06, *SE* = 0.09, *t(328)* = −0.61, *p* = .542; 3-month follow-up: *β* = −0.05, *SE* = 0.10, *t(142)* = −0.46, *p* = .649). After applying Bonferroni correction for multiple testing, this pattern remained the same.

**Fig 9:**
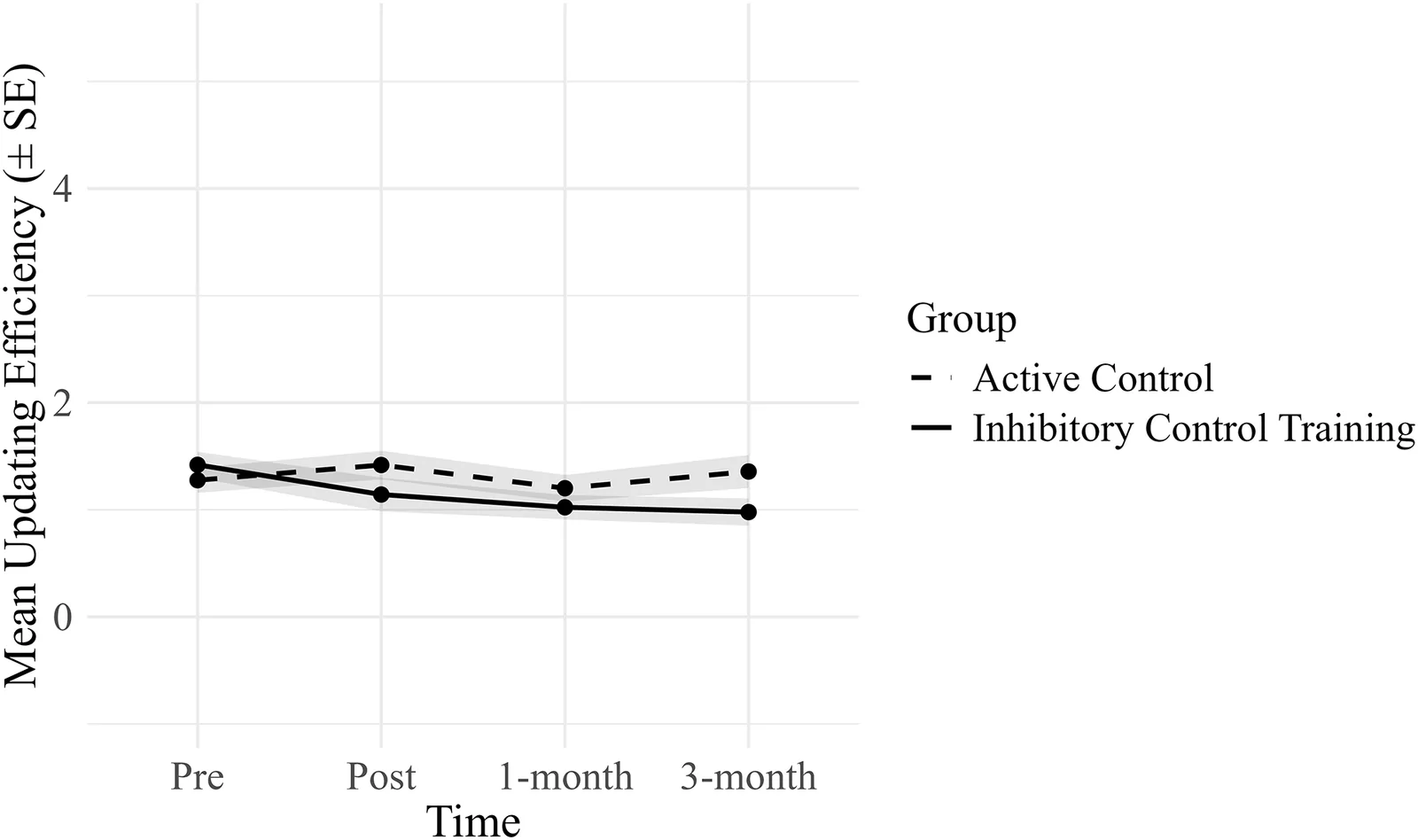
Mean Updating Efficiency per Training Group Across Time – ITT. *SE* = Standard Error.

Finally, for updating efficiency, Group × Time interactions were significant at post-training (*β* = −0.39, *SE* = 0.17, *t(299)* = −2.29, *p* = .023, *β*_*std*_  = −0.37, 95% CI = [−0.69, −0.05]) and at the 1-month follow-up (*β* = −0.49, *SE* = 0.20, *t(140)* = −2.47, *p* = .015, *β*_*std*_  = −0.47, 95% CI = [−0.85, −0.10]). Fig 9 illustrates the mean updating efficiency per group across time. Simple slope analyses showed significant decreases from pre-training at both time points in the inhibitory control group (post-training: *β* = −0.27, *SE* = 0.12, *t(296)* = −2.31, *p* = .022; 3-month follow-up: *β* = −0.44, *SE* = 0.14, *t(135)* = −3.21, *p* = .002), but not for the active control group (post-training: *β* = 0.11, *SE* = 0.12, *t(301)* = 0.95, *p* = .345; 3-month follow-up: *β* = 0.05, *SE* = 0.14, *t(146)* = 0.34, *p* = .733). However, after applying Bonferroni correction, only the decrease in the outcome from pre-training at 3-month follow-up remained significant.

### Training effects on emotional symptoms

To test H2, we conducted separate LMMs with anxiety, worry, depression, rumination and math anxiety as dependent variables in the ITT sample. For all emotional outcomes, likelihood-ratio tests indicated that the model including both a random intercept and a random slope for Time fit the data better than a random-intercept-only model. Accordingly, the more complex model was used in the analyses. The model coefficients, standard errors, degrees of freedom, test statistics, *p*-values, and effect sizes (including 95% CI) of each model are presented in Table 4.

**Table 4:** LMM results for emotional measures – ITT sample.

Outcome	Predictors	*ß*	*SE*	*df*	*t*	*p*	*ß*_*std*_ [95% CI]
Anxiety	Intercept	33.11	2.64	131.16	12.54	< .001	0.18 [−0.07, 0.42]
	Group	−6.78	3.72	131.15	−1.82	.071	−0.32 [−0.66, 0.03]
	Timepoint 2	−1.11	1.42	325.49	−0.78	.434	−0.05 [−0.18, 0.08]
	Timepoint 3	−0.79	1.58	296.03	−0.50	.619	−0.04 [−0.18, 0.11]
	Timepoint 4	−0.08	1.82	135.98	−0.05	.964	−0.004 [−0.17, 0.16]
	Group × Timepoint 2	−0.26	2.00	325.48	−0.13	.898	−0.01 [−0.20, 0.17]
	Group × Timepoint 3	−0.25	2.23	296.02	−0.11	.912	−0.01 [−0.22, 0.19]
	Group × Timepoint 4	1.50	2.56	135.97	0.59	.559	0.07 [−0.17, 0.31]
Worry	Intercept	17.32	1.18	133.15	14.73	< .001	0.06 [−0.19, 0.31]
	Group	−0.96	1.66	133.14	−0.58	.562	−0.10 [−0.45, 0.25]
	Timepoint 2	0.13	0.70	342.70	0.18	.855	0.01 [−0.13, 0.16]
	Timepoint 3	−0.64	0.82	260.84	−0.78	.434	−0.07 [−0.24, 0.10]
	Timepoint 4	0.77	1.00	132.41	0.77	.443	0.08 [−0.13, 0.29]
	Group × Timepoint 2	0.42	0.98	342.69	0.43	.666	0.05 [−0.16, 0.25]
	Group × Timepoint 3	0.34	1.16	260.83	0.29	.770	0.04 [−0.21, 0.28]
	Group × Timepoint 4	−1.75	1.40	132.40	−1.25	.215	−0.19 [−0.48, 0.11]
Depression	Intercept	10.84	0.92	137.15	11.76	< .001	0.20 [−0.05, 0.45]
	Group	−2.30	1.30	137.15	−1.78	.078	−0.32 [−0.66, 0.03]
	Timepoint 2	−0.74	0.61	319.72	−1.21	.229	−0.10 [−0.27, 0.06]
	Timepoint 3	−0.77	0.67	307.72	−1.15	.251	−0.11 [−0.29, 0.07]
	Timepoint 4	−0.34	0.76	136.92	−0.46	.650	−0.05 [−0.25, 0.16]
	Group × Timepoint 2	0.21	0.86	319.72	0.24	.812	0.03 [−0.20, 0.26]
	Group × Timepoint 3	0.38	0.94	307.72	0.41	.685	0.05 [−0.20, 0.31]
	Group × Timepoint 4	0.68	1.07	136.92	0.64	.523	0.09 [−0.19, 0.38]
Rumination	Intercept	15.33	1.30	140.04	11.82	< .001	0.18 [−0.06, 0.42]
	Group	−1.99	1.83	140.04	−1.09	.278	−0.19 [−0.53, 0.15]
	Timepoint 2	−1.21	0.95	336.03	−1.28	.203	−0.11 [−0.29, 0.06]
	Timepoint 3	−1.54	1.10	275.17	−1.40	.162	−0.15 [−0.35, 0.06]
	Timepoint 4	0.13	1.31	133.10	0.10	.920	0.01 [−0.23, 0.26]
	Group × Timepoint 2	−0.77	1.34	336.03	−0.58	.566	−0.07 [−0.32, 0.18]
	Group × Timepoint 3	0.32	1.55	275.17	0.20	.839	0.03 [−0.26, 0.32]
	Group × Timepoint 4	−1.34	1.84	133.10	−0.73	.468	−0.13 [−0.47, 0.22]
Math Anxiety	Intercept	9.61	0.58	142.97	16.63	<.001	0.11 [-0.14, 0.36]
	Group	−0.39	0.82	143.70	−0.48	.631	−0.09 [−0.44, 0.27]
	Timepoint 2	0.16	0.44	310.73	0.37	.710	0.04 [−0.15, 0.23]
	Timepoint 3	−0.52	0.47	323.11	−1.12	.265	−0.12 [−0.32, 0.09]
	Timepoint 4	−0.57	0.52	139.17	−1.11	.267	−0.13 [−0.35, 0.10]
	Group × Timepoint 2	−0.75	0.62	311.87	−1.20	.230	−0.17 [−0.43, 0.10]
	Group × Timepoint 3	0.21	0.66	323.27	0.32	.747	0.05 [−0.24, 0.33]
	Group × Timepoint 4	−0.03	0.73	139.75	−0.04	.970	−0.01 [−0.32, 0.31]

Timepoint 1 = Pre-training; Timepoint 2 = Post-training, Timepoint 3 = 1-month follow-up, Timepoint 4 = 3-month follow-up. CI = Confidence interval.

The LMM examining anxiety in the ITT sample showed that none of the main effects (Group or Time) nor any of the Group × Time interaction effects were statistically significant (all *p*-values > .071), indicating that anxiety levels did not differ between groups at baseline and showed no differential change over time. The same null pattern of non-significant main and interaction effects was observed for worry, depression, rumination, and math anxiety. Full model estimates for all emotional outcomes are presented in Table 4, and a descriptive table including the mean outcomes for each group at each timepoint can be found in the supplementary materials (Supplementary Table 1 in S1 File).

### Training effects on maths achievement

To evaluate the theoretical plausibility of transfer effects, we first examined the baseline association between inhibitory control and mathematics achievement. A significant small-to-moderate association was found between baseline inhibitory control effectiveness and maths achievement (*r* = 0.21; *p* = .020), and a moderate association was observed for inhibitory control efficiency (*r* = .35; *p* = < .001), providing support for the theoretical rationale linking inhibitory control and mathematics performance.

To test H3, we then conducted a LMM in the ITT sample with math achievement as the outcome. The likelihood-ratio test indicated that the random intercept-only model provided a better fit, and this model was thus retained. The results are presented in Table 5. No significant main effect of Group was found, indicating no difference between the inhibitory control training group and the active control group at baseline (*t(144)* = 0.63, *p* = .533, *β*_*std*_  = 0.11, 95% CI = [−0.24, 0.46]). Main effects of Time were found at post-training (*t(362)* = 2.87, *p* = .004, *β*_*std*_  = 0.17, 95% CI = [0.05, 0.29]) and at 3-months follow-up (*t(362)* = 7.46, *p* < .001, *β*_*std*_  = 0.45, 95% CI = [0.33, 0.57]). There were no significant Group × Time interactions at any time point, suggesting no differential effects of inhibitory control training on math achievement over time.

**Table 5:** LMM results for maths achievement – ITT sample.

Outcome	Predictors	*ß*	*SE*	*df*	*t*	*p*	*ß*_*std*_ [95% CI]
Maths	Intercept	488.91	1.86	144.33	262.68	< .001	−0.21 [−0.46, 0.04]
Achievement	Group	1.64	2.62	144.00	0.63	.533	0.11 [−0.24, 0.46]
	Timepoint 2	2.53	0.88	362.10	2.87	.004	0.17 [0.05, 0.29]
	Timepoint 3	1.65	0.88	362.10	1.87	.063	0.11 [−0.01, 0.23]
	Timepoint 4	6.58	0.88	362.10	7.46	< .001	0.45 [0.33, 0.57]
	Group × Timepoint 2	−1.11	1.24	362.06	−0.90	.370	−0.08 [−0.24, 0.09]
	Group × Timepoint 3	−1.47	1.24	362.06	−1.19	.237	−0.10 [−0.27, 0.07]
	Group × Timepoint 4	−1.19	1.24	362.06	−0.96	.336	−0.08 [−0.25, 0.08]

Timepoint 1 = Pre-training; Timepoint 2 = Post-training, Timepoint 3 = 1-month follow-up, Timepoint 4 = 3-month follow-up. CI = Confidence interval.

### Per-protocol analyses

In addition to the ITT analyses, we repeated the analyses described above in a per-protocol sample (*n* = 96), excluding those who completed less than 11 training sessions. This approach was guided by previous research indicating that approximately 11–15 sessions targeting executive functions are generally needed to detect improvements in cognitive control and reductions in emotional symptoms in children [25]. The main findings are summarized below; full results can be found in Supplementary Tables 3–5 in S1 File. Overall, significant Group × Time interactions were found for all executive outcome measures except for inhibitory efficiency. Regarding the emotional outcome measures and the math achievement outcomes, no significant Group × Time interactions were found.

**Fig 10:**
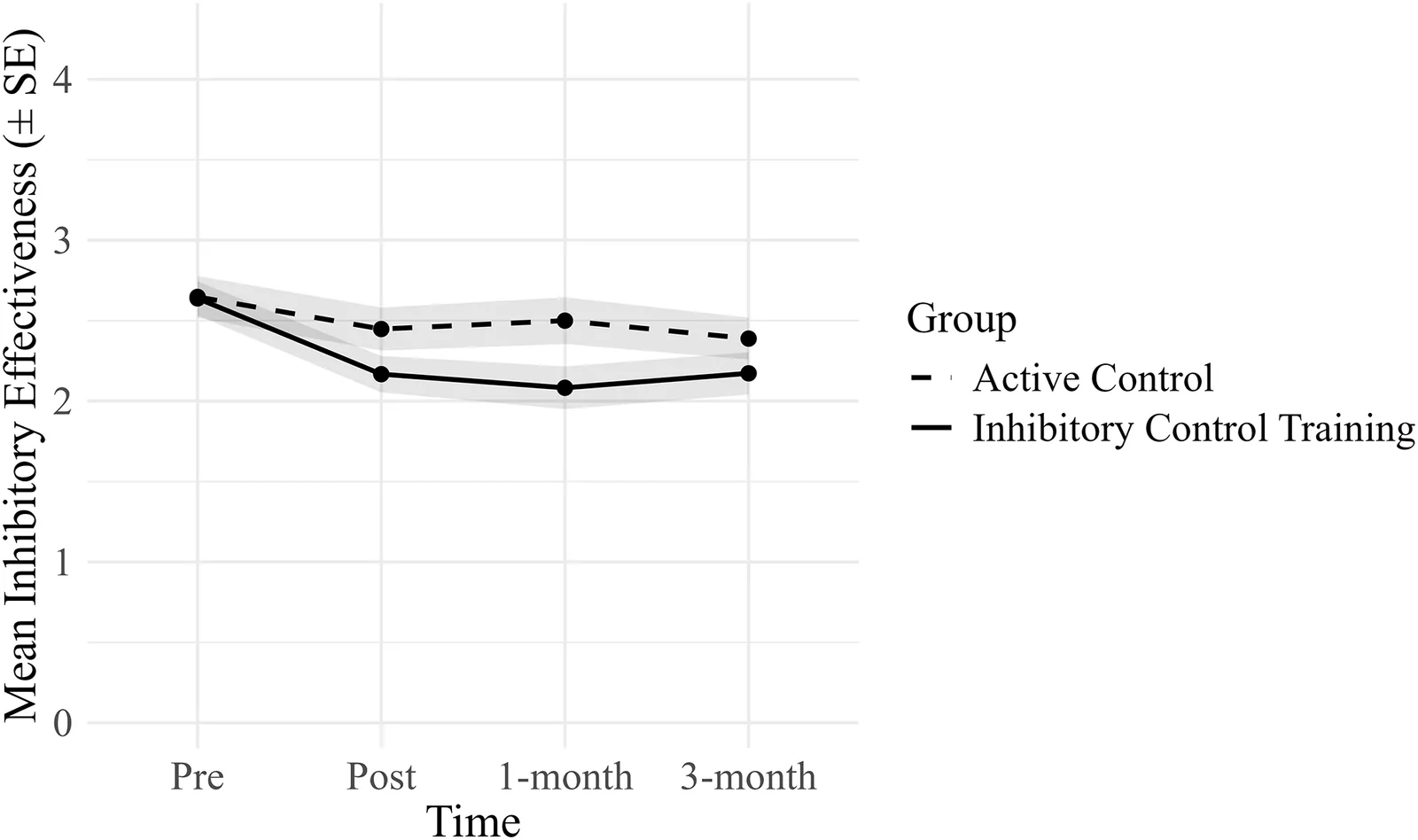
Mean Inhibitory Effectiveness per Training Group Across Time – per-protocol sample. *SE* = Standard Error.

For inhibitory effectiveness, the LMM revealed a significant Group × Time interaction at 1-month follow-up (*β* = −0.39, *SE* = 0.19, *t*(276) = −2.02, *p* = .045, *β*_*std*_ = −0.43, CI = [−0.86, −0.01]), indicating that changes in inhibitory effectiveness depended on the training group. Fig 10 illustrates mean inhibitory effectiveness per group across timepoints. Follow-up simple slope analyses revealed a significant decrease in the outcome from pre-training to the 1-month follow-up in the inhibitory control condition (*β* = −0.57, *SE* = 0.14, *t*(276) = −4.14, *p* < .001), and not in the active control condition (*β* = −0.17, *SE* = 0.14, *t*(277) = −1.27, *p* = .205). The effec*t*s remained significant after applying Bonferroni correction.

**Fig 11:**
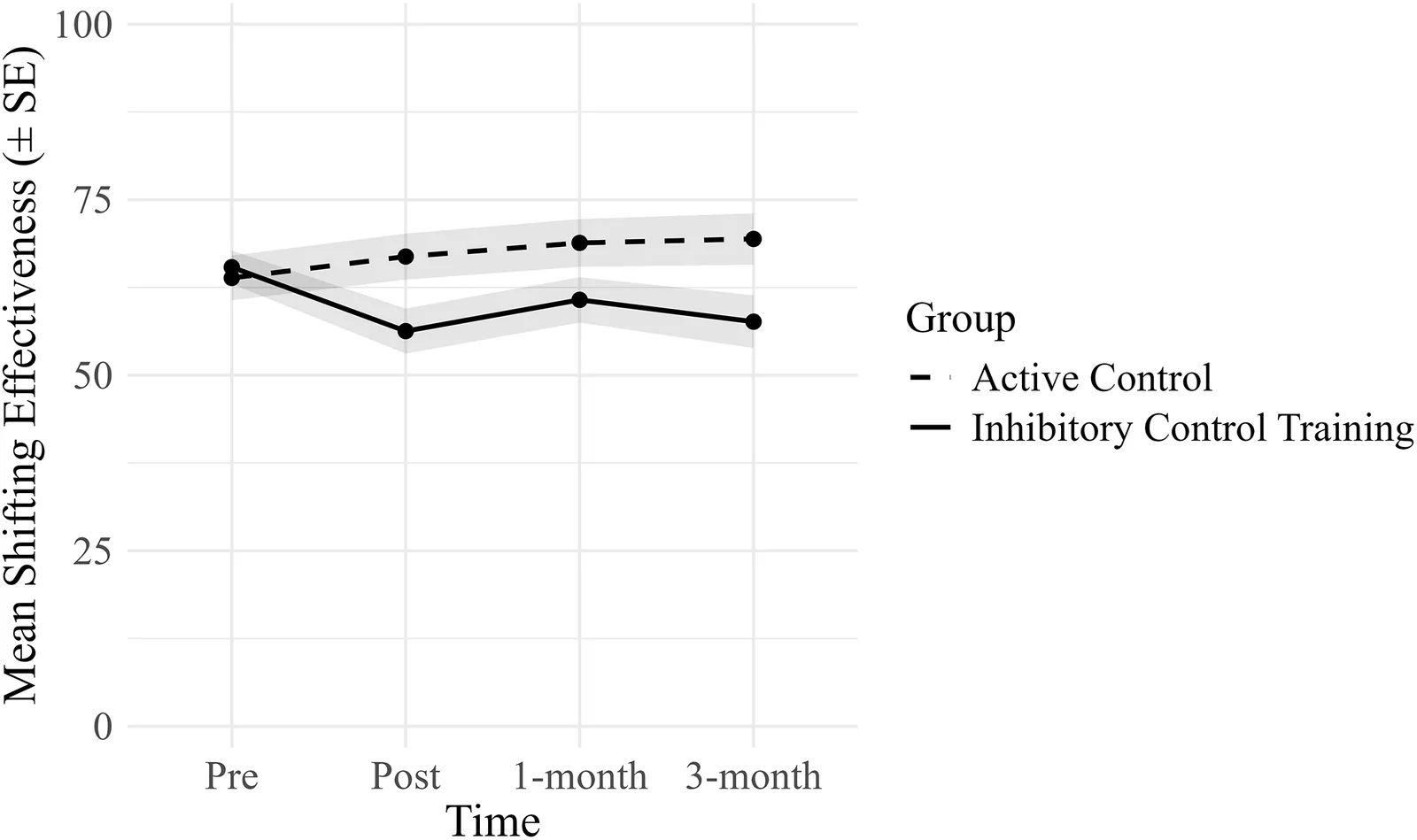
Mean Shifting Effectiveness per Training Group Across Time – per-protocol sample. *SE* = Standard Error.

Next, for shifting effectiveness, significant Group × Time interactions were observed at all three time points (post-training: *β* = −12.00, *SE* = 4.28, *t*(233) = −2.80, *p* = .005, *β*_*std*_ = −0.52, CI = [−0.89, −0.16]; 1-month follow-up: *β* = −9.20, *SE* = 4.62, *t*(251) = −1.99, *p* = .048, *β*_*std*_ = −0.40, CI = [−0.79, −0.005]; 3-month follow-up: *β* = −13.45, *SE* = 4.96, *t*(103) = −2.71, *p* = .008, *β*_*std*_ = −0.58, CI = [−1.01, −0.16]), indicating differential changes in shifting efficiency over time between groups (Fig 11). Follow-up simple slope analyses showed that within the inhibitory control training group, shifting effectiveness decreased significantly from pre- to post-training (*β* = −8.94, *SE* = 3.03, *t*(233) = −2.96, *p* = .003) and from pre-training to the 3-month follow-up (*β* = −7.60, *SE* = 3.50, *t*(102) = −2.17, *p* = .032), whereas no significant changes were observed at the 1-month follow-up (*β* = −4.73, *SE* = 3.27, *t*(251) = −1.45, *p* = .149). In contrast, the active control group showed no significant changes at any time point (post-training: *β* = 3.06, *SE* = 3.03, *t*(233) = 1.01, *p* = .313; 1-month follow-up: *β* = 4.46, *SE* = 3.27, *t*(251) = 1.37, *p* = .173; 3-month follow-up: *β* = 5.85, *SE* = 3.52, *t*(103) = 1.66, *p* = .100). After applying Bonferroni correction, only the decrease from pre- to post-training in the inhibitory control training group remained statistically significant.

**Fig 12:**
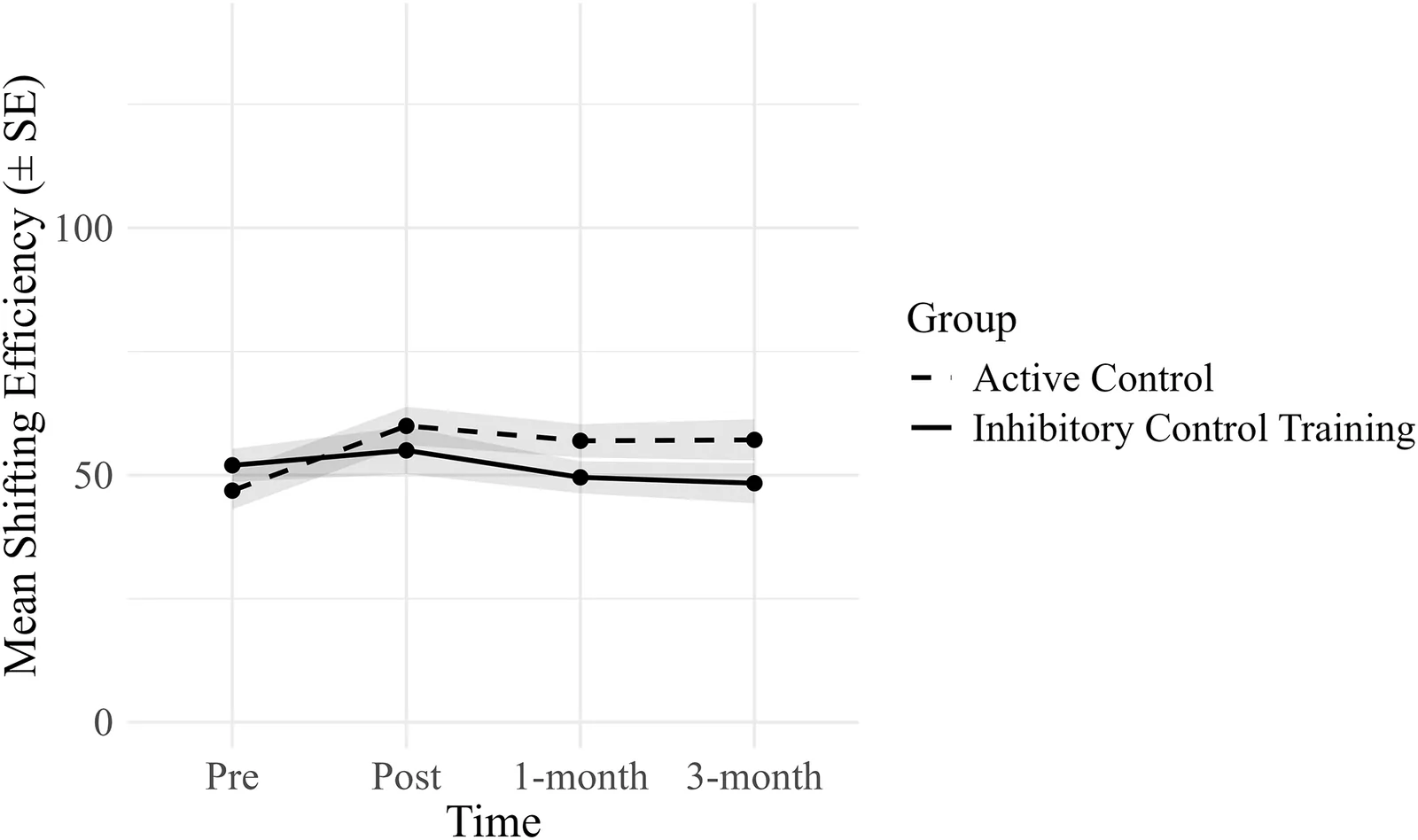
Mean Shifting Efficiency per Training Group Across Time – per-protocol sample. *SE* = Standard Error.

The LMM for shifting efficiency revealed significant Group × Time interactions at the 1-month (*β* = −12.86, *SE* = 6.01, *t*(245) = −2.14, *p* = .026, *β*_*std*_ = 0.39, CI = [0.08, 0.70]) and 3-month follow-up (*β* = −14.66, *SE* = 6.60, *t*(102) = −2.22, *p* = .029, *β*_*std*_ = −0.39, CI = [0.05, 0.73]; see Fig 12). Simple slopes analyses however, indicated no significant changes over time within the inhibitory control training group, either from pre-training to the 1-month follow-up (*β* = −2.36, *SE* = 4.25, *t*(245) = −0.56, *p* = .579) or from pre-training to the 3-month follow-up (*β* = −4.12, *SE* = 4.65, *t*(102) = −0.88, *p* = .378). In contrast, participants in the active control group exhibited significant increases in shifting efficiency from pre-training to the 1-month follow-up (*β* = 10.50, *SE* = 4.25, *t*(245) = 2.47, *p* = .014) and from pre-training to the 3-month follow-up (*β* = 10.54, *SE* = 4.68, *t*(103) = 2.25, *p* = .026). These effects, however, did not remain statistically significant after Bonferroni correction for multiple comparisons.

**Fig 13:**
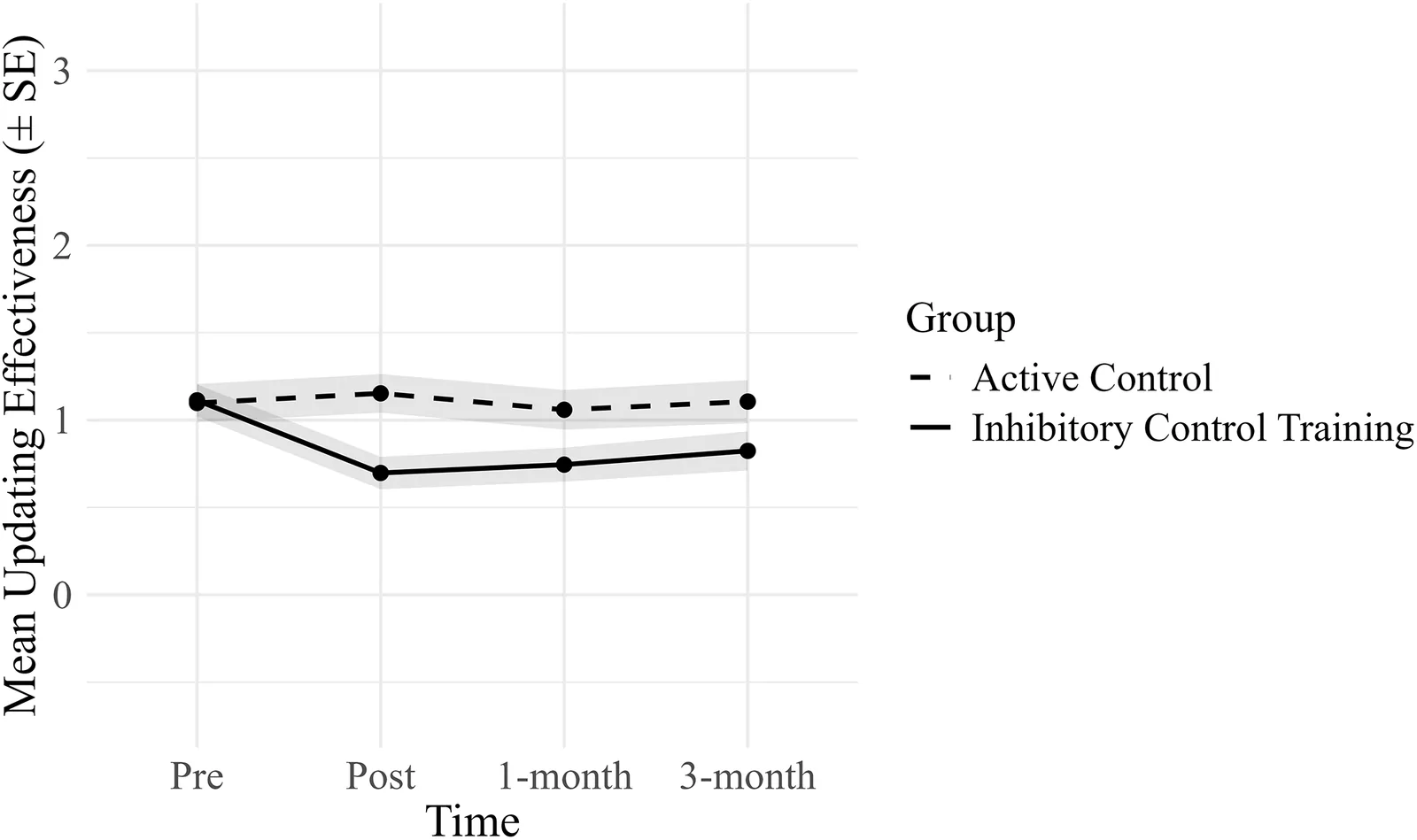
Mean Updating Effectiveness per Training Group Across Time – per-protocol sample. SE  =  Standard Error.

Next, regarding updating effectiveness, Group × Time interactions were significant at 1-month follow-up (*β* = −0.45, *SE* = 0.14, *t*(239) = −3.29, *p* = .001, *β*_*std*_ = −0.60, CI = [−0.95, −0.24]) and at 3-month follow-up (*β* = −0.31, *SE* = 0.15, *t*(247) = −2.10, *p* = .037, *β*_*std*_ = −0.41, CI = [−0.79, −0.03]). Fig 13 shows the mean updating effectiveness per group across time. Follow-up simple slope analyses revealed significant decreases within the inhibitory control training group from pre- to post-training (*β* = −0.41, *SE* = 0.10, *t*(239) = −4.23, *p* < .001) and from pre-training to the 1-month follow-up (*β* = −0.36, *SE* = 0.10, *t*(246) = −3.52, *p* < .001), whereas no significan*t* changes in updating effectiveness were observed in the active control group over the same intervals (pre- to post-training: *β* = 0.04, *SE* = 0.10, *t*(239) = 0.41, *p* = .685; pre-training *t*o 1-month follow-up: *β* = −0.06, *SE* = 0.10, *t*(248) = −0.54, *p* = .889). Applying Bonferroni correction did no*t* alter this pattern.

**Fig 14:**
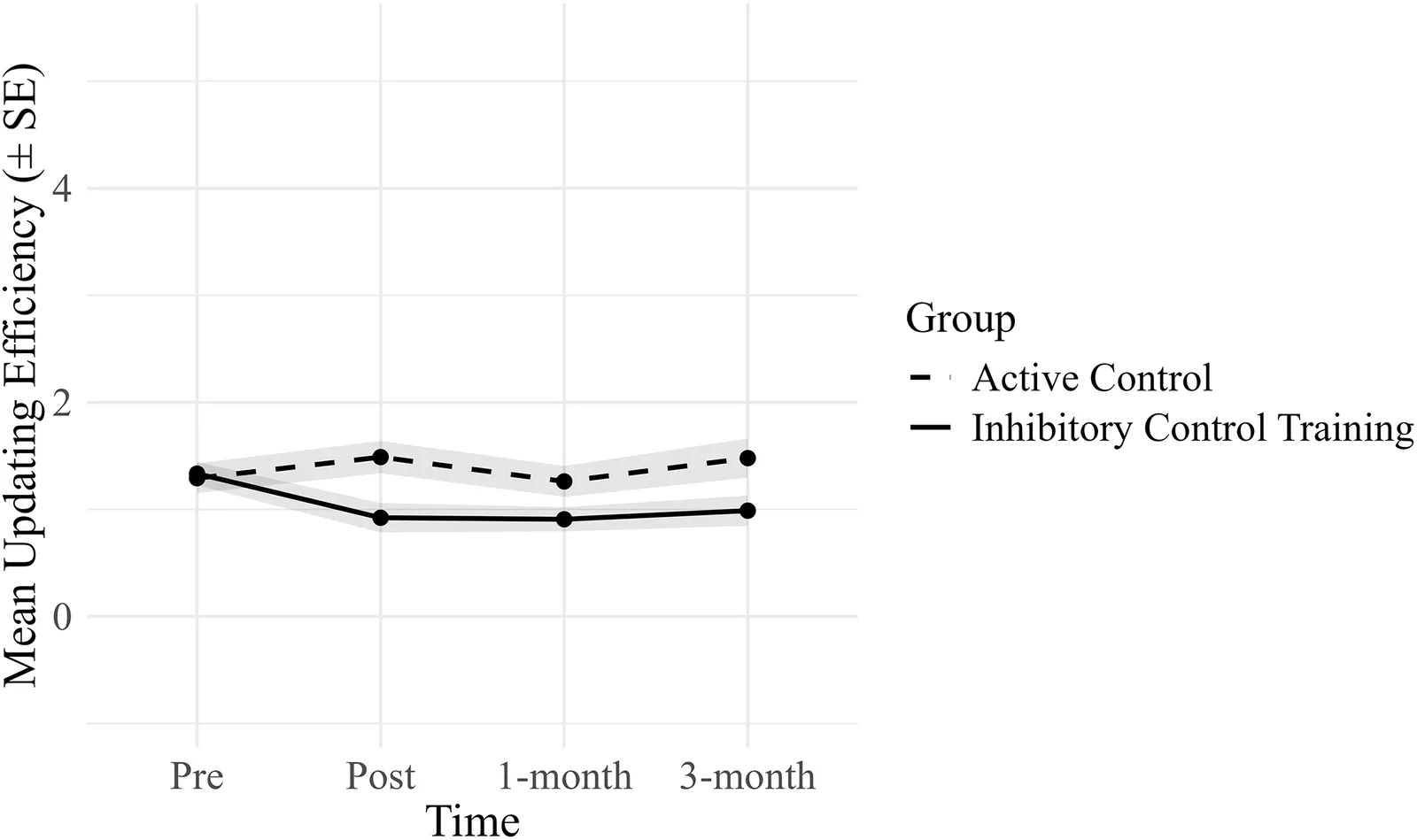
Mean Updating Efficiency per Training Group Across Time – per-protocol sample. SE  =  Standard Error.

Finally, the LMM for updating efficiency revealed significant Group × Time interactions at post-training (*β* = −0.58, *SE* = 0.19, *t*(235) = −3.14, *p* = .002, *β*_*std*_ = −0.59, CI = [−0.96, −0.22]) and at the 3-month follow-up (*β* = −0.50, *SE* = 0.22, *t*(108) = −2.24, *p* = .027, *β*_*std*_ = −0.51, CI = [−0.96, −0.06]) time points (Fig 14). Follow-up simple slope analyses showed that within the inhibitory control training group, updating efficiency decreased significantly from pre- to post-training (*β* = −0.41, *SE* = 0.13, *t*(234) = −3.12, *p* = .002) and from pre-training to the 3-month follow-up (*β* = −0.35, *SE* = 0.16, *t*(104) = −2.22, *p* = .028). In contrast, no significant changes in updating efficiency were observed in the active control group from pre- to post-training (*β* = 0.18, *SE* = 0.13, *t*(236) = 1.34, *p* = .182) or from pre-training to 3-month follow-up (*β* = 0.16, *SE* = 0.16, *t*(112) = 0.97, *p* = .335). After applying Bonferroni correction for multiple comparisons, only the decrease from pre- to post-training in the inhibitory control training group remained statistically significant.

## Discussion

Our randomized controlled trial tested the efficacy of a gamified inhibitory control, delivered to a universal sample of children in school settings, to reduce emotional vulnerability in middle childhood. In line with the published and pre-registered protocol [48], we examined whether adaptive Go/No-Go training could produce near and far cognitive transfer effects and, in turn, lead to reductions in emotional vulnerability and improvements in math achievement across four timepoints. Although children in the inhibitory control training group demonstrated clear learning on the training task itself, the hypothesized near and far transfer effects, as well as downstream emotional and academic benefits, were not observed. Our findings diverged from predictions derived from attentional control theory [10], and studies conducted with children linking inhibitory control with anxiety [34] and math performance [79] and demonstrating that inhibitory control training can reduce emotional symptoms such as anxiety and depression [39]. Importantly, however, the results provide valuable insight into the boundary conditions under which inhibitory control training may, or may not, be sufficient to produce transferable cognitive or emotional change in middle childhood.

### Training task performance, near and far transfer effects

Children assigned to the inhibitory control training condition showed reliable improvement in performance on the adaptive Go/No-Go task across training sessions, evidenced by reductions in false alarm rates over time. This pattern indicates successful engagement with training, confirms that children acquired the specific response contingencies required by the paradigm and provides a necessary manipulation check for the cognitive training intervention. Despite this task-specific learning, no evidence of near transfer to untrained measures of inhibitory control (i.e., non-adaptive Go/No-Go) were observed, nor were there improvements in broader executive functions (far transfer), particularly, shifting (Colour Shape task) and updating (*N*-back task).

One possible explanation for this pattern concerns the task-specific nature of cognitive training effects. Executive functions, including inhibitory control, are not unitary constructs but comprise partially dissociable subcomponents [80]. While our inhibitory control training task and the near transfer task both targeted prepotent response inhibition (adaptive and non-adaptive Go/No-Go, respectively), transfer effects may be even more subcomponent-specific than anticipated. Subtle task-specific differences such as adaptive versus non-adaptive formats, response rules, and stimulus presentation may have reduced the functional overlap required for transfer. These nuanced variations could have activated slightly different inhibitory processes, thereby limiting the generalization of training effects. A further interpretation aligns with longstanding critiques in the cognitive training literature. Large-scale and methodologically rigorous studies have repeatedly shown that training one executive function does not reliably translate to untrained tasks, whether within or across cognitive domains in both children [46,69] and adults [74,81,82].

Neither of these explanations, nonetheless, account for the unexpected pattern whereby cognitive performance of children in the inhibitory control training group declined at post-training or follow-up compared to baseline, whereas performance of those in the active control group remained stable. Although initially counterintuitive, this pattern was consistently observed across multiple executive function indices, including inhibitory and updating effectiveness, and updating efficiency, and was evident in both ITT and per-protocol analyses. It is possible that the inhibitory control training task may have imposed suboptimal cognitive demands across the intervention period, that is, it was too difficult. While the task was adaptive, repeated exposure may have encouraged a heightened emphasis on cautionary responding and error avoidance. When subsequently faced with the non-adaptive assessment tasks under speeded conditions, children in the inhibitory control training group may have retained this conservative response strategy, resulting in slower RTs and potentially increased error rates when instructed to work quickly. In contrast, children in the active control condition, who completed the less demanding visual identification task, may not have experienced comparable shifts in response strategy, allowing their cognitive performance to remain more stable across assessment timepoints.

### Training effects on emotional symptoms

Contrary to our predictions, we observed no effects of inhibitory control training on anxiety, worry, depression or rumination. These findings contrast those of Shanok et al. [39] who reported reductions in anxiety and depression following inhibitory control training, and other studies who deployed commercial working memory training in middle childhood [37,38] and studies with adults at-risk for depression who have observed reductions in depressive symptoms following working memory training [18]. Yet, our findings concur with Ganesan et al. [42] who reported no changes in internalizing symptoms following inhibitory control training, and Schweizer et al. [35] who noted no changes in rumination following working memory training in adolescents.

The most likely reason for these null results is that, while cognitive transfer may be a necessary precondition for emotional change, it is not sufficient in itself. Our systematic review suggested that improvements in cognitive control are typically required for downstream emotional benefits [25]; however, recent evidence indicated that even when near transfer is observed, this does not necessarily translate into reductions in internalizing symptoms. For example, Ganesan et al. [42] reported improvements in inhibitory control following training but no corresponding changes in emotional outcomes. It is possible, therefore, that additional factors, such as the extent of transfer, the nature of the training, or the characteristics of the sample, may moderate whether cognitive transfer gains translate into emotional benefits.

Prior studies demonstrating beneficial effects of cognitive control training have often focussed on clinical and at-risk samples [19–22,24], whereas our training was delivered to a universal sample of children in school settings. As such, our non-select sample had relatively low baseline anxiety, worry, depression and rumination. Specifically, possible depression scores ranged 0–30, yet mean baseline depression in our sample was 8.72, and possible rumination scores ranged 0–39 and mean baseline rumination was 12.80. As such, it is likely that children in our sample with low initial symptom severity had limited room for improvement.

Finally, our hypothesis that inhibitory control training would beneficially impact math anxiety was also not supported. Given that no prior studies have examined domain-specific anxiety changes (e.g., math anxiety) in cognitive control paradigms, this result is difficult to reconcile. In line with the explanation for other internalizing symptoms, it is possible that math anxiety scores ranging 5–25 with a sample mean 9.02 reflects compressed or low math anxiety which was underpowered for change.

### Training effect on math achievement

All children continued receiving regular math instruction during the intervention, however, we expected to see greater achievements for children in the inhibitory control group relative to controls. Notwithstanding the education setting which potentially diluted the unique contribution of the training, both groups improved over time and there were no group differences. Again, the most logical explanation for these null results are likely due to the lack of cognitive transfer. Our findings, however, align with Wilkinson et al. [46], who implemented inhibitory control training embedded within national mathematics and science curricula. While their intervention did not enhance math achievement, science performance improved in the cognitive training group relative to controls, suggesting that domain-specific integration may be crucial for academic transfer. Supporting this view, a recent study involving children aged 7–12 years reported significant improvements in math performance following cognitive control training that incorporated math-specific content [83]. Notably, the gains may not be attributed solely to cognitive or academic training elements, but rather to their synergistic combination.

The inclusion of mathematics achievement as an exploratory outcome was based on evidence linking inhibitory control with mathematics performance during middle childhood [47]. Consistent with this rationale, additional baseline analyses also indicated small-to-moderate positive associations between inhibitory control and math achievement (see Results section). Although inhibitory control may influence mathematics outcomes through both cognitive and emotional pathways, the absence of significant improvements in emotional symptoms in the present study, together with absence of training effects on math achievement, provides limited support for indirect effects via reduced anxiety or math anxiety. More broadly, the absence of transfer effects across both emotional and academic outcomes raises questions regarding the conditions under which inhibitory control training generalises beyond the trained task when delivered to universal samples of children.

## Directions for future research

We followed our published protocol, our results were consistent across ITT and per-protocol analyses, yet our hypotheses were not upheld. Although the present study had a larger sample than many previous child cognitive training studies [24,35,37–39], it may still have been underpowered to detect small effects, particularly in the per-protocol analyses where approximately 22% of participants completed fewer than 11 training sessions. The preregistered sample size was intended to detect effects in the small-to-moderate/moderate range; however, smaller transfer effects on emotional outcomes may have gone undetected. This is especially relevant given the universal prevention design and relatively low baseline symptom levels, which may have restricted the scope for measurable improvement. Accordingly, the null findings should be interpreted as evidence against moderate intervention effects under the present conditions, rather than definitive evidence that inhibitory control training has no effect on emotional outcomes in children.

The following recommendations are based on the limitations of our work. Future studies may benefit from pre-selecting children based on (sub)clinical anxiety, worry or depression, or cognitive control impairments or elevated repetitive negative thinking. Studies warrant balancing the contextual factors of the cognitive training task with the cognitive assessments used at baseline, post-training and follow-up. Particularly, ensuring the training and assessment tasks are matched for interest/enjoyment (e.g., include game elements in all tasks) and ensure tasks are of corresponding difficulty/complexity (e.g., consistency of response strategies). Furthermore, including children in the development phase of tasks and training paradigms (e.g., through focus groups) may assist with understanding response strategies, dosage and engagement.

## Conclusion

The present study examined the effects of gamified inhibitory control training delivered to typically developing children in middle childhood on cognitive, emotional, and academic outcomes. Despite assessing a comprehensive range of outcome measures, including several previously shown to be sensitive to cognitive training, the intervention did not yield transferable benefits beyond task-specific learning. These findings align with a growing body of evidence highlighting the limits of cognitive training transfer and suggest that suboptimal or diminishing cognitive demands may constrain the efficacy of universal inhibitory control interventions. Future research should investigate alternative training designs, including tasks that sustain optimal cognitive challenge or target specific at-risk populations, to illuminate meaningful transfer across educational and community contexts.

## Supporting information

S1 File Supplemental Table 1. Mean Outcomes per Group per Timepoint – ITT. Note. Mean (SD). Efficiency measures are multiplied by 1,000 to improve interpretability. Supplemental Table 2. Mean Outcomes per Group per Timepoint – per-protocol sample. Supplemental Table 3. LMM results for cognitive performance measures – per-protocol sample. Supplemental Table 4. LMM results for emotional measures – per-protocol sample. Supplemental Table 5. LMM results for math achievement – per-protocol sample. R-package version information.
(DOCX)
