# Supplementary material for: Evaluating the effects of inhibitory control training as a universal prevention strategy for internalizing complaints in middle childhood
Source: PLoS One. 2026 Sep 21;21(9):e0358051. doi: 10.1371/journal.pone.0358051 (PMC13592700; doi:10.1371/journal.pone.0358051)
Supplement: Supplementary file 1 [file pone.0358051.s001.docx]

**Supplementary materials**

**Supplemental Table 1. Mean Outcomes per Group per Timepoint – ITT**

|  | **Pre-training** | | **Post-training** | |
| --- | --- | --- | --- | --- |
|  |  |  |  |  |
|  | **Inhibitory** | **Active** | **Inhibitory** | **Active** |
|  | **Control Training** | **Control** | **Control Training** | **Control** |
| **Emotional measures** |  |  |  |  |
| Anxiety | 25.90 (18.28) | 33.47 (23.50) | 24.97 (17.02) | 32.00 (22.28) |
| Worry | 16.30 (8.92) | 17.47 (9.16) | 16.90 (8.03) | 17.44 (9.68) |
| Depression | 8.53 (6.33) | 10.84 (7.47) | 8.00 (6.78) | 10.10 (7.44) |
| Rumination | 13.34 (10.00) | 15.33 (10.23) | 11.35 (9.96) | 14.11 (10.05) |
| Math Anxiety | 9.26 (4.15) | 9.61 (4.41) | 8.63 (4.03) | 9.77 (5.60) |
|  |  |  |  |  |
| **Cognitive measures** |  |  |  |  |
| Inhibitory Effectiveness | 2.59 (0.71) | 2.59 (0.88) | 2.19 (0.82) | 2.44 (0.87) |
| Inhibitory Efficiency | 4.37 (1.28) | 4.45 (1.76) | 3.64 (1.49) | 4.20 (1.75) |
| Shifting Effectiveness | 63.26 (18.70) | 64.49 (21.06) | 57.46 (22.50) | 66.02 (22.95) |
| Shifting Efficiency | 52.29 (23.20) | 48.17 (26.38) | 56.81 (31.87) | 58.01 (27.35) |
| Updating Effectiveness | 1.15 (0.71) | 1.06 (0.72) | 0.85 (0.81) | 1.10 (0.78) |
| Updating Efficiency | 1.28 (0.91)) | 1.42 (0.96) | 1.14 (1.23) | 1.42 (1.04) |
|  |  |  |  |  |
| **Math achievement**  **measure** | |  |  |  |
| Math Achievement | 490.55 (13.24) | 488.87 (16.76) | 491.97 (12.91) | 491.44 (15.68) |
|  |  |  |  |  |

*Note.* Mean (*SD*). Efficiency measures are multiplied by 1,000 to improve interpretability.

|  | **1 month follow-up** | | **3 month follow-up** | |  |  |  |  |
| --- | --- | --- | --- | --- | --- | --- | --- | --- |
|  |  |  |  |  |  |  |  |  |
|  | **Inhibitory** | **Active** | **Inhibitory** | **Active** |  |  |  |  |
|  | **Control Training** | **Control** | **Control Training** | **Control** |  |  |  |  |
| **Emotional measures** |  |  |  |  |  |  |  |  |
| Anxiety | 25.31 (19.17) | 32.33 (23.55) | 27.76 (21.28) | 33.03 (23.34) |  |  |  |  |
| Worry | 16.05 (10.26) | 16.67 (9.38) | 15.37 (9.54) | 18.08 (9.61) |  |  |  |  |
| Depression | 8.15 (6.70) | 10.07 (8.08) | 8.87 (7.10) | 10.49 (8.21) |  |  |  |  |
| Rumination | 12.11 (11.36) | 13.79 (10.38) | 12.13 (10.66) | 15.46 (11.55) |  |  |  |  |
| Math Anxiety | 8.90 (4.54) | 9.08 (4.90) | 8.61 (3.96) | 9.03 (4.59) |  |  |  |  |
|  |  |  |  |  |  |  |  |  |
| **Cognitive measures** |  |  |  |  |  |  |  |  |
| Inhibitory Effectiveness | 2.12 (0.93) | 2.57 (1.01) | 2.13 (0.95) | 2.37 (0.91) |  |  |  |  |
| Inhibitory Efficiency | 3.42 (1.58) | 4.15 (1.95) | 3.41 (1.64) | 3.94 (1.69) |  |  |  |  |
| Shifting Effectiveness | 61.31 (23.12) | 67.27 (24.32) | 56.77 (27.57) | 68.81 (24.29) |  |  |  |  |
| Shifting Efficiency | 50.24 (22.03) | 55.99 (25.54) | 49.21 (28.08) | 56.48 (27.14) |  |  |  |  |
| Updating Effectiveness | 0.84 (0.70) | 1.01 (0.77) | 0.83 (0.83) | 1.03 (0.84) |  |  |  |  |
| Updating Efficiency | 1.02 (0.89) | 1.20 (0.97) | 0.98 (1.00) | 1.36 (1.20) |  |  |  |  |
|  |  |  |  |  |  |  |  |  |
| **Math achievement**  **measure** | | | | | |  |  |  |
| Math Achievement | 490.73 (14.32) | 490.56 (16.41) | 495.94 (12.09) | 495.49 (14.26) |  |  |  |  |
|  |  |  |  |  |  |  |  |  |
| *Note.* Mean (*SD*). Efficiency measures are multiplied by 1,000 to improve interpretability. | | | | | | | | |

**Supplemental Table 2. Mean Outcomes per Group per Timepoint – per-protocol sample**

|  | **Pre-training** | | | | **Post-training** | | | |  |  |
| --- | --- | --- | --- | --- | --- | --- | --- | --- | --- | --- |
|  | **Inhibitory** | | **Active** | | **Inhibitory** | | **Active** | |  |  |
|  | **Control Training** | | **Control** | | **Control Training** | | **Control** | |  |  |
| **Emotional measures** |  | |  | |  | |  | |  |  |
| Anxiety | 24.64 (17.64) | | 30.13 (21.32) | | 24.31 (18.00) | | 29.04 (19.60) | |  |  |
| Worry | 15.23 (8.42) | | 16.13 (8.31) | | 15.92 (7.98) | | 15.92 (8.98) | |  |  |
| Depression | 8.38 (6.04) | | 9.98 (7.34) | | 7.35 (6.10) | | 9.21 (6.93) | |  |  |
| Rumination | 12.48 (10.21) | | 14.25 (9.72) | | 10.63 (10.30) | | 13.10 (9.37) | |  |  |
| Math Anxiety | 9.60 (4.52) | | 9.19 (4.25) | | 8.81 (4.33) | | 9.29 (5.51) | |  |  |
|  |  | |  | |  | |  | |  |  |
| **Cognitive measures** |  | |  | |  | |  | |  |  |
| Inhibitory Effectiveness | 2.64 (0.74) | | 2.65 (0.90) | | 2.17 (0.78) | | 2.45 (0.93) | |  |  |
| Inhibitory Efficiency | 4.31 (1.26) | | 4.50 (1.77) | | 3.49 (1.37) | | 4.26 (1.82) | |  |  |
| Shifting Effectiveness | 65.39 (16.48) | | 63.86 (22.03) | | 56.29 (22.17) | | 66.90 (22.71) | |  |  |
| Shifting Efficiency | 51.97 (23.15) | | 46.85 (26.20) | | 54.99 (33.06) | | 59.94 (26.80) | |  |  |
| Updating Effectiveness | 1.11 (0.64) | | 1.10 (0.75) | | 0.70 (0.64) | | 1.15 (0.77) | |  |  |
| Updating Efficiency | 1.33 (0.75) | | 1.29 (0.96) | | 0.92 (0.95) | | 1.49 (1.04) | |  |  |
|  |  | |  | |  | |  | |  |  |
| **Math achievement measure** | | |  | |  | |  | |  |  |
| Math Achievement | 488.94 (13.83) | | 490.53 (17.05) | | 490.67 (13.08) | | 493.56 (15.47) | |  |  |
|  |  | |  | |  | |  | |  |  |
|  | | **1-month follow-up** | | | | **3-month follow-up** | | | |  |
|  | | **Inhibitory** | | **Active** | | **Inhibitory** | | **Active** | |  |
|  | | **Control Training** | | **Control** | | **Control Training** | | **Control** | |  |
| **Emotional measures** | |  | |  | |  | |  | |  |
| Anxiety | | 25.25 (20.78) | | 29.56 (21.98) | | 27.94 (22.13) | | 29.10 (19.74) | |  |
| Worry | | 15.25 (10.95) | | 15.96 (9.48) | | 14.94 (9.66) | | 16.63 (9.56) | |  |
| Depression | | 7.94 (6.41) | | 9.29 (7.78) | | 8.96 (6.99) | | 9.65 (8.02) | |  |
| Rumination | | 12.27 (11.72) | | 12.75 (8.83) | | 11.63 (10.85) | | 14.23 (11.05) | |  |
| Math Anxiety | | 9.31 (4.85) | | 8.98 (4.99) | | 8.79 (4.10) | | 8.75 (4.59) | |  |
|  | |  | |  | |  | |  | |  |
| **Cognitive measures** | |  | |  | |  | |  | |  |
| Inhibitory Effectiveness | | 2.08 (0.92) | | 2.50 (1.00) | | 2.17 (0.91) | | 2.39 (0.90) | |  |
| Inhibitory Efficiency | | 3.32 (1.58) | | 4.11 (2.00) | | 3.46 (1.64) | | 3.97 (1.64) | |  |
| Shifting Effectiveness | | 60.74 (22.39) | | 68.85 (23.62) | | 57.63 (26.05) | | 69.41 (25.30) | |  |
| Shifting Efficiency | | 49.53 (22.46) | | 56.93 (23.40) | | 48.35 (28.11) | | 57.12 (29.00) | |  |
| Updating Effectiveness | | 0.75 (0.67) | | 1.06 (0.79) | | 0.82 (0.77) | | 1.11 (0.85) | |  |
| Updating Efficiency | | 0.91 (0.78) | | 1.26 (1.00) | | 0.99 (0.98) | | 1.48 (1.26) | |  |
|  | |  | |  | |  | |  | |  |
| **Math achievement measure** | | | |  | |  | |  | |  |
| Math Achievement | | 489.31 (14.62) | | 492.48 (16.23) | | 494.90 (12.70) | | 497.19 (14.40) | |  |
|  | |  | |  | |  | |  | |  |
| *Note.* Mean (*SD*). | | | | | | | | | |  |

**Supplemental Table 3. LMM results for cognitive performance measures – per-protocol sample**

| **Outcome** | **Predictors** | ***ß*** | ***SE*** | ***df*** | ***t*** | ***p*** | ***β_std_ [95% CI]*** |
| --- | --- | --- | --- | --- | --- | --- | --- |
|  |  |  |  |  |  |  |  |
| Inhibitory | Intercept | 2.66 | 0.13 | 235.49 | 20.64 | < .001 | 0.31 [0.03, 0.59] |
| Effectiveness | Group | -0.03 | 0.18 | 235.49 | -0.16 | .871 | -0.03 [-0.43, 0.36] |
|  | Timepoint 2 | -0.21 | 0.14 | 274.90 | -1.56 | .120 | -0.23 [-0.53, 0.06] |
|  | Timepoint 3 | -0.17 | 0.14 | 276.53 | -1.27 | .205 | -0.19 [-0.49, 0.11] |
|  | Timepoint 4 | -0.26 | 0.14 | 275.43 | -1.94 | .054 | -0.29 [-0.59, 0.004] |
|  | Group × Timepoint 2 | -0.25 | 0.19 | 274.90 | -1.31 | .190 | -0.28 [-0.69, 0.14] |
|  | Group × Timepoint 3 | -0.39 | 0.19 | 276.26 | -2.02 | .045 | -0.43 [-0.86, -0.01] |
|  | Group × Timepoint 4 | -0.19 | 0.19 | 275.17 | -1.01 | .315 | -0.21 [-0.63, 0.20] |
|  |  |  |  |  |  |  |  |
| Inhibitory | Intercept | 4.54 | 0.24 | 204.69 | 18.98 | < .001 | 0.37 [0.09, 0.64] |
| Efficiency | Group | -0.25 | 0.34 | 204.69 | -0.75 | .455 | -0.15 [-0.55, 0.24] |
|  | Timepoint 2 | -0.28 | 0.23 | 274.39 | -1.22 | .225 | -0.17 [-0.44, 0.10] |
|  | Timepoint 3 | -0.46 | 0.24 | 275.70 | -1.94 | .053 | -0.27 [-0.55, 0.003] |
|  | Timepoint 4 | -0.55 | 0.23 | 274.82 | -2.36 | .019 | -0.33 [-0.60, -0.05] |
|  | Group × Timepoint 2 | -0.52 | 0.33 | 274.39 | -1.58 | .116 | -0.31 [-0.69, 0.08] |
|  | Group × Timepoint 3 | -0.55 | 0.33 | 275.48 | -1.66 | .098 | -0.33 [-0.72, 0.06] |
|  | Group × Timepoint 4 | -0.28 | 0.33 | 274.61 | -0.85 | .398 | -0.16 [-0.55, 0.22] |
|  |  |  |  |  |  |  |  |
| Shifting | Intercept | 63.85 | 3.10 | 125.63 | 20.63 | < .001 | 0.01 [-0.25, 0.28] |
| Effectiveness | Group | 1.38 | 4.38 | 125.63 | 0.32 | .752 | 0.06 [-0.31, 0.43] |
|  | Timepoint 2 | 3.06 | 3.03 | 232.96 | 1.01 | .313 | 0.13 [-0.13, 0.39] |
|  | Timepoint 3 | 4.47 | 3.27 | 250.75 | 1.37 | .173 | 0.19 [-0.09, 0.47] |
|  | Timepoint 4 | 5.85 | 3.52 | 103.28 | 1.66 | .100 | 0.25 [-0.05, 0.55] |
|  | Group × Timepoint 2 | -12.00 | 4.28 | 232.96 | -2.80 | .005 | -0.52 [-0.89, -0.16] |
|  | Group × Timepoint 3 | -9.20 | 4.62 | 250.75 | -1.99 | .048 | -0.40 [-0.79, 0.005] |
|  | Group × Timepoint 4 | -13.45 | 4.96 | 102.70 | -2.71 | .008 | -0.58 [-1.01, -0.16] |
|  |  |  |  |  |  |  |  |
| Shifting | Intercept | 46.69 | 3.96 | 121.91 | 11.79 | < .001 | -0.24 [-0.53, 0.05] |
| Efficiency | Group | 5.78 | 5.60 | 121.91 | 1.03 | .304 | 0.21 [-0.19, 0.62] |
|  | Timepoint 2 | 13.26 | 3.87 | 232.49 | 3.43 | < .001 | 0.49 [0.21, 0.78] |
|  | Timepoint 3 | 10.50 | 4.25 | 244.70 | 2.47 | .014 | 0.39 [0.08, 0.70] |
|  | Timepoint 4 | 10.54 | 4.68 | 102.84 | 2.25 | .026 | 0.39 [0.05, 0.73] |
|  | Group × Timepoint 2 | -10.73 | 5.47 | 232.49 | -1.96 | .051 | -0.40 [-0.80, 0.001] |
|  | Group × Timepoint 3 | -12.86 | 6.01 | 244.70 | -2.14 | .034 | -0.48 [-0.92, -0.04] |
|  | Group × Timepoint 4 | -14.66 | 6.60 | 102.24 | -2.22 | .029 | -0.54 [-1.03, -0.06] |
|  |  |  |  |  |  |  |  |
| Updating | Intercept | 1.11 | 0.10 | 121.48 | 10.91 | < .001 | 0.19 [-0.08, 0.45] |
| Effectiveness | Group | -0.01 | 0.14 | 121.48 | -0.04 | .966 | -0.01 [-0.39, 0.37] |
|  | Timepoint 2 | 0.04 | 0.10 | 239.34 | 0.41 | .685 | 0.05 [-0.20, 0.30] |
|  | Timepoint 3 | -0.06 | 0.10 | 248.13 | -0.54 | .588 | -0.08 [-0.35, 0.20] |
|  | Timepoint 4 | -0.01 | 0.11 | 108.68 | -0.09 | .933 | -0.01 [-0.31, 0.29] |
|  | Group × Timepoint 2 | -0.45 | 0.14 | 239.36 | -3.29 | .001 | -0.60 [-0.95, -0.24] |
|  | Group × Timepoint 3 | -0.31 | 0.15 | 247.21 | -2.10 | .037 | -0.41 [-0.79, -0.03] |
|  | Group × Timepoint 4 | -0.27 | 0.16 | 108.09 | -1.70 | .092 | -0.36 [-0.79, 0.06] |
|  |  |  |  |  |  |  |  |
| Updating | Intercept | 1.30 | 0.13 | 123.89 | 9.71 | < .001 | 0.09 [-0.17, 0.36] |
| Efficiency | Group | 0.03 | 0.19 | 123.01 | 0.16 | .872 | 0.03 [-0.34, 0.41] |
|  | Timepoint 2 | 0.18 | 0.13 | 236.27 | 1.34 | .182 | 0.18 [-0.08, 0.44] |
|  | Timepoint 3 | -0.04 | 0.14 | 238.31 | -0.29 | .774 | -0.04 [-0.32, 0.24] |
|  | Timepoint 4 | 0.16 | 0.16 | 112.07 | 0.97 | .335 | 0.16 [-0.16, 0.48] |
|  | Group × Timepoint 2 | -0.58 | 0.19 | 234.93 | -3.14 | .002 | -0.59 [-0.96, -0.22] |
|  | Group × Timepoint 3 | -0.39 | 0.20 | 238.04 | -1.95 | .052 | -0.40 [-0.80, 0.003] |
|  | Group × Timepoint 4 | -0.50 | 0.22 | 107.88 | -2.24 | .027 | -0.51 [-0.96, -0.06] |
|  |  |  |  |  |  |  |  |
| *Note.* Timepoint 1 = Pre-training; Timepoint 2 = Post-training, Timepoint 3 = 1-month follow-up; Timepoint 4 = 3-month follow-up. CI = Confidence interval. | | | | | | | |

**Supplemental Table 4. LMM results for emotional measures – per-protocol sample**

| **Outcome** | **Predictors** | ***ß*** | ***SE*** | ***df*** | ***t*** | ***p*** | ***β_std_ [95%* CI*]*** |
| --- | --- | --- | --- | --- | --- | --- | --- |
|  |  |  |  |  |  |  |  |
| Anxiety | Intercept | 29.74 | 2.79 | 103.23 | 10.66 | < .001 | 0.11 [-0.16, 0.38] |
|  | Group | -4.54 | 3.95 | 103.23 | -1.15 | .253 | -0.23 [-0.61, 0.16] |
|  | Timepoint 2 | -0.70 | 1.60 | 244.91 | -0.44 | .662 | -0.03 [-0.19, 0.12] |
|  | Timepoint 3 | -0.18 | 1.72 | 246.20 | -0.10 | .917 | -0.01 [-0.18, 0.16] |
|  | Timepoint 4 | -0.64 | 1.90 | 108.00 | -0.34 | .738 | -0.03 [-0.22, 0.15] |
|  | Group × Timepoint 2 | -0.19 | 2.26 | 244.91 | -0.09 | .932 | -0.01 [-0.23, 0.21] |
|  | Group × Timepoint 3 | 0.22 | 2.43 | 246.20 | 0.09 | .927 | 0.01 [-0.23, 0.25] |
|  | Group × Timepoint 4 | 3.37 | 2.68 | 108.00 | 1.26 | .212 | 0.17 [-0.09, 0.43] |
|  |  |  |  |  |  |  |  |
| Worry | Intercept | 15.97 | 1.23 | 104.93 | 12.96 | < .001 | 0.02 [-0.24, 0.29] |
|  | Group | -0.64 | 1.74 | 104.93 | -0.37 | .712 | -0.07 [-0.45, 0.30] |
|  | Timepoint 2 | -0.05 | 0.77 | 262.19 | -0.06 | .949 | -0.01 [-0.17, 0.16] |
|  | Timepoint 3 | -0.01 | 0.89 | 211.84 | -0.01 | .993 | -0.001 [-0.19, 0.19] |
|  | Timepoint 4 | 0.66 | 1.06 | 103.87 | 0.62 | .536 | 0.07 [-0.16, 0.30] |
|  | Group × Timepoint 2 | 0.64 | 1.09 | 262.19 | 0.59 | .554 | 0.07 [-0.16, 0.30] |
|  | Group × Timepoint 3 | -0.06 | 1.26 | 211.84 | -0.05 | .960 | -0.01 [-0.28, 0.26] |
|  | Group × Timepoint 4 | -1.04 | 1.50 | 103.87 | -0.70 | .489 | -0.11 [-0.44, 0.21] |
|  |  |  |  |  |  |  |  |
| Depression | Intercept | 9.98 | 0.99 | 108.00 | 10.10 | < .001 | 0.16 [-0.12, 0.44] |
|  | Group | -1.60 | 1.40 | 108.00 | -1.15 | .254 | -0.23 [-0.62, 0.16] |
|  | Timepoint 2 | -0.77 | 0.69 | 251.91 | -1.11 | .268 | -0.11 [-0.31, 0.09] |
|  | Timepoint 3 | -0.69 | 0.77 | 232.04 | -0.89 | .374 | -0.10 [-0.32, 0.12] |
|  | Timepoint 4 | -0.33 | 0.89 | 105.45 | -0.38 | .707 | -0.05 [-0.30, 0.20] |
|  | Group × Timepoint 2 | -0.25 | 0.98 | 251.91 | -0.26 | .799 | -0.04 [-0.31, 0.24] |
|  | Group × Timepoint 3 | 0.25 | 1.09 | 232.04 | 0.23 | .819 | 0.04 [-0.27, 0.34] |
|  | Group × Timepoint 4 | 0.92 | 1.25 | 105.45 | 0.73 | .466 | 0.13 [-0.22, 0.48] |
|  |  |  |  |  |  |  |  |
| Rumination | Intercept | 14.25 | 1.42 | 108.09 | 10.01 | < .001 | 0.15 [-0.12, 0.43] |
|  | Group | -1.77 | 2.01 | 108.09 | -0.88 | .381 | -0.17 [-0.56, 0.21] |
|  | Timepoint 2 | -1.15 | 1.01 | 256.69 | -1.13 | .259 | -0.11 [-0.31, 0.08] |
|  | Timepoint 3 | -1.50 | 1.15 | 222.50 | -1.31 | .193 | -0.15 [-0.37, 0.07] |
|  | Timepoint 4 | -0.02 | 1.34 | 104.33 | -0.02 | .988 | -0.002 [-0.26, 0.26] |
|  | Group × Timepoint 2 | -0.71 | 1.43 | 256.69 | -0.49 | .621 | -0.07 [-0.34, 0.21] |
|  | Group × Timepoint 3 | 1.29 | 1.62 | 222.50 | 0.80 | .427 | 0.13 [-0.19, 0.44] |
|  | Group × Timepoint 4 | -0.83 | 1.90 | 104.33 | -0.44 | .662 | -0.08 [-0.45, 0.28] |
|  |  |  |  |  |  |  |  |
| Math | Intercept | 9.19 | 0.67 | 158.43 | 13.65 | < .001 | 0.02 [-0.26, 0.31] |
| Anxiety | Group | 0.34 | 0.95 | 159.59 | 0.36 | .721 | 0.07 [-0.33, 0.48] |
|  | Timepoint 2 | 0.10 | 0.54 | 281.01 | 0.19 | .848 | 0.02 [-0.21, 0.25] |
|  | Timepoint 3 | -0.21 | 0.54 | 281.01 | -0.39 | .701 | -0.04 [-0.28, 0.19] |
|  | Timepoint 4 | -0.44 | 0.54 | 281.01 | -0.81 | .420 | -0.09 [-0.32, 0.14] |
|  | Group × Timepoint 2 | -0.82 | 0.77 | 281.15 | -1.07 | .287 | -0.18 [-0.50, 0.15] |
|  | Group × Timepoint 3 | -0.01 | 0.77 | 281.15 | -0.01 | .992 | -0.002 [-0.33, 0.32] |
|  | Group × Timepoint 4 | -0.30 | 0.77 | 281.15 | -0.39 | .697 | -0.06 [-0.39, 0.26] |
|  |  |  |  |  |  |  |  |
| *Note.* Timepoint 1 = Pre-training; Timepoint 2 = Post-training, Timepoint 3 = 1-month follow-up; Timepoint 4 = 3-month follow-up. CI = Confidence interval. | | | | | | | |

**Supplemental Table 5. LMM results for math achievement – per-protocol sample**

| **Outcome** | **Predictors** | ***ß*** | ***SE*** | ***df*** | ***t*** | ***p*** | ***β_std_ [95%* CI*]*** |
| --- | --- | --- | --- | --- | --- | --- | --- |
|  |  |  |  |  |  |  |  |
| Math | Intercept | 490.55 | 2.13 | 112.20 | 230.59 | < .001 | -0.11 [-0.39, 0.17] |
| Achievement | Group | -1.61 | 3.01 | 111.87 | -0.54 | .593 | -0.11 [-0.51, 0.29] |
|  | Timepoint 2 | 3.01 | 1.01 | 281.10 | 2.99 | .003 | 0.20 [0.07, 0.34] |
|  | Timepoint 3 | 1.93 | 1.01 | 281.10 | 1.91 | .057 | 0.13 [-0.004, 0.26] |
|  | Timepoint 4 | 6.64 | 1.01 | 281.10 | 6.59 | < .001 | 0.45 [0.31, 0.58] |
|  | Group × Timepoint 2 | -1.28 | 1.42 | 281.06 | -0.90 | .367 | -0.09 [-0.27, 0.10] |
|  | Group × Timepoint 3 | -1.55 | 1.42 | 281.06 | -1.09 | .275 | -0.10 [-0.29, 0.08] |
|  | Group × Timepoint 4 | -0.68 | 1.42 | 281.06 | -0.48 | .633 | -0.05 [-0.23, 0.14] |
|  |  |  |  |  |  |  |  |
| *Note.* Timepoint 1 = Pre-training; Timepoint 2 = Post-training, Timepoint 3 = 1-month follow-up; Timepoint 4 = 3-month follow-up. CI = Confidence interval. | | | | | | | |

**R-package version information**

R version 4.5.2 (2025-10-31 ucrt)

Platform: x86_64-w64-mingw32/x64

Running under: Windows 11 x64 (build 26100)

Matrix products: default

LAPACK version 3.12.1

locale:

[1] LC_COLLATE=Dutch_Belgium.utf8 LC_CTYPE=Dutch_Belgium.utf8	LC_MONETARY=Dutch_Belgium.utf8 LC_NUMERIC=C	LC_TIME=Dutch_Belgium.utf8

time zone: Europe/Paris

tzcode source: internal

attached base packages:

[1] stats	graphics grDevices utils	datasets methods	base

other attached packages:

[1] extrafont_0.20	emmeans_2.0.0	see_0.12.0	performance_0.15.2 reghelper_1.1.2	r2glmm_0.1.3	lmerTest_3.1-3	lme4_1.1-37	Matrix_1.7-4	ggpubr_0.6.2	lubridate_1.9.4	forcats_1.0.1	stringr_1.6.0	purrr_1.1.0	readr_2.1.5	tidyr_1.3.1	tibble_3.3.0	ggplot2_4.0.0	tidyverse_2.0.0	dplyr_1.1.4	haven_2.5.5

loaded via a namespace (and not attached):

[1] tidyselect_1.2.1	farver_2.1.2	S7_0.2.0	fastmap_1.2.0	digest_0.6.37	estimability_1.5.1 timechange_0.3.0	lifecycle_1.0.4	magrittr_2.0.4	compiler_4.5.2	rlang_1.1.6	tools_4.5.2	yaml_2.3.10	knitr_1.50	ggsignif_0.6.4	labeling_0.4.3	plyr_1.8.9	RColorBrewer_1.1-3 abind_1.4-8	withr_3.0.2	numDeriv_2016.8-1.1 datawizard_1.3.0	grid_4.5.2	afex_1.5-0	extrafontdb_1.1	scales_1.4.0	MASS_7.3-65	insight_1.4.3	cli_3.6.5	mvtnorm_1.3-3	crayon_1.5.3	rmarkdown_2.30	reformulas_0.4.2	generics_0.1.4	rstudioapi_0.17.1	reshape2_1.4.5	tzdb_0.5.0	minqa_1.2.8	splines_4.5.2	parallel_4.5.2	vctrs_0.6.5	boot_1.3-32	carData_3.0-5	car_3.1-3	patchwork_1.3.2	hms_1.1.4	ggrepel_0.9.6	rstatix_0.7.3	Formula_1.2-5	glue_1.8.0	nloptr_2.2.1	stringi_1.8.7	gtable_0.3.6	pillar_1.11.1	htmltools_0.5.8.1	R6_2.6.1	Rdpack_2.6.4	evaluate_1.0.5	lattice_0.22-7	rbibutils_2.4	backports_1.5.0	broom_1.0.10	Rcpp_1.1.0	Rttf2pt1_1.3.14	nlme_3.1-168	mgcv_1.9-3	xfun_0.54	pkgconfig_2.0.3
